# Monitoring extracellular ion and metabolite dynamics with recombinant nanobody-fused biosensors

**DOI:** 10.1016/j.isci.2022.104907

**Published:** 2022-08-10

**Authors:** Sandra Burgstaller, Teresa R. Wagner, Helmut Bischof, Sarah Bueckle, Aman Padamsey, Desiree Frecot, Philipp D. Kaiser, David Skrabak, Roland Malli, Robert Lukowski, Ulrich Rothbauer

**Affiliations:** 1 Pharmaceutical Biotechnology, Eberhard Karls University Tübingen, Markwiesenstrasse 55, 72770 Reutlingen, Germany; 2 Department of Pharmacology, Toxicology and Clinical Pharmacy, Institute of Pharmacy, University of Tübingen, Auf der Morgenstelle 8, 72076 Tuebingen, Germany; 3 NMI Natural and Medical Sciences Institute at the University of Tübingen, Markwiesenstrasse 55, 72770 Reutlingen, Germany; 4 Gottfried Schatz Research Center, Molecular Biology and Biochemistry, Medical University of Graz, Neue Stiftingtalstraße 6/6, 8010 Graz, Austria; 5 BioTechMed-Graz, Mozartgasse 12/II, 8010 Graz, Austria

**Keywords:** Sensor, Optical imaging, Biochemistry

## Abstract

Ion and analyte changes in the tumor microenvironment (TME) alter the metabolic activity of cancer cells, promote tumor cell growth, and impair anti-tumor immunity. Consequently, accurate determination and visualization of extracellular changes of analytes in real time is desired. In this study, we genetically combined FRET-based biosensors with nanobodies (Nbs) to specifically visualize and monitor extracellular changes in K^+^, pH, and glucose on cell surfaces. We demonstrated that these Nb-fused biosensors quantitatively visualized K^+^ alterations on cancer and non-cancer cell lines and primary neurons. By implementing a HER2-specific Nb, we generated functional K^+^ and pH sensors, which specifically stained HER2-positive breast cancer cells. Based on the successful development of several Nb-fused biosensor combinations, we anticipate that this approach can be readily extended to other biosensors and will open new opportunities for the study of extracellular analytes in advanced experimental settings.

## Highlights

• Generation of recombinant nanobody-fused FRET biosensors
• Nb-fused biosensors specifically bind targets on the outer surface of various cells
• Cellular bound Nb-biosensors visualize extracellular analyte changes in real time

## Graphical abstract

**Figure:**
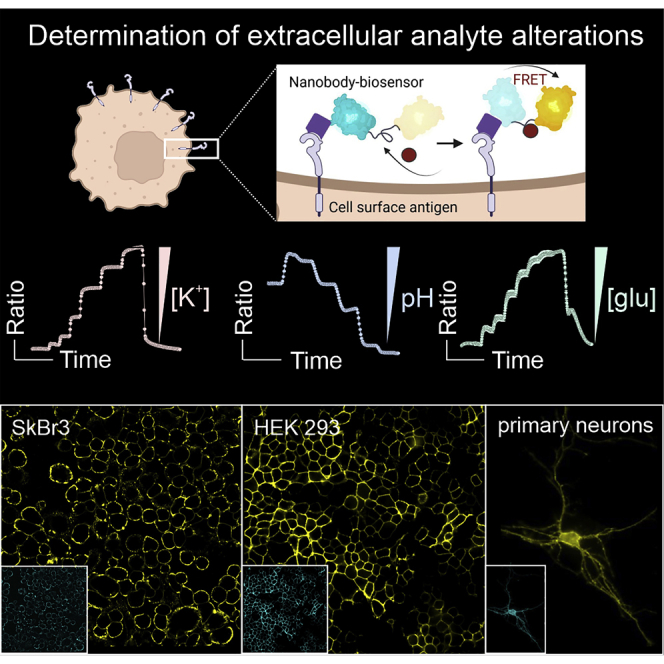

## Introduction

The tumor microenvironment (TME) represents a highly specialized niche where tumor-associated-stromal cells, immune cells, blood and lymphatic vessels create an oncogenic milieu featuring nutrients, growth factors, cytokines, and abnormal alterations of intra- and extracellular ion and metabolite levels (Anderson and Simon, 2020). Cancer cell metabolism and proliferation are heavily influenced by these microenvironmental factors and their interactions (Costanza et al., 2019; Huntington et al., 2022; Pedersen et al., 2017; Soroceanu et al., 1999). In this context, the Warburg effect describes that cancer cells prefer glycolysis to oxidative phosphorylation despite the presence of molecular oxygen (O_2_) (Vander Heiden et al., 2009). This metabolic switch, also referred to as aerobic glycolysis, yields lactate production and subsequent secretion, thereby significantly acidifying the TME toward a pH of 6.5 or lower (Gallagher et al., 2008; Vander Heiden et al., 2009). This in turn affects cell and tissue organization. For example, acidic pH is known to trigger extracellular matrix (ECM) restructuring, leading to loss of ECM integrity (Busco et al., 2010; Webb et al., 2011) which facilitates the spread of cancer and tumor dissemination (Frantz et al., 2007). Associated with this phenomenon, the altered metabolism of cancer cells provides increased glucose uptake to meet the increased energy demand during proliferation (Han et al., 2015; Yang et al., 2013). Hence, the extracellular glucose concentration ([GLU]_ex_) represents a growth-determining factor (Cao et al., 2007; Xu et al., 2015). Moreover, the TME not only promotes cancer growth by metabolically reprogramming but also by modulating responses of the immune system (Eil et al., 2016). As a result of improper vascularization, solid tumor growth is associated with necrotic cell death within the tumor core. During necrosis, high intracellular K^+^ levels are released into the TME, affecting the function of effector T-cells, ultimately causing cancer cells to escape the immune system (Eil et al., 2016). In summary, the interplay of such extracellular changes causes the development of a hostile environment that is presumed to impair the efficacy of chemotherapeutic agents (Jähde et al., 1990; Thews et al., 2011; Yang et al., 2020).

Considering this, real-time monitoring of ionic and metabolic changes in the extracellular milieu could broaden our understanding of the bidirectional crosstalk between tumor cells and stromal cells. By maintaining a cellular resolution of these intra-tumoral signaling factors, such strategies might also help to unravel unknown mechanisms that promote stromal cell recruitment by the tumor, cancer metabolism, and cell malignancy.

Förster resonance energy transfer (FRET)-based biosensors are powerful tools for measuring ions and analytes at the cellular level (Bischof et al., 2019; Burgstaller et al., 2022; Depaoli et al., 2019). These biosensors usually consist of two fluorescent proteins acting as FRET donor and acceptor, respectively, linked by an analyte-binding domain (Depaoli et al., 2019). The design of FRET-based biosensors enables reporting of changes in the analyte by increasing or decreasing FRET efficiency, a process that is fast, highly dynamic, and reversible (Depaoli et al., 2019). Most currently applied FRET-based sensors visualize intracellular analyte fluctuations. However, this requires genetic cell manipulation either by transient or stable transfection of the respective FRET biosensor, which limits their application because of drawbacks such as low transfection rates of primary cells or alteration of cell metabolic activities (Fiszer-Kierzkowska et al., 2011; Jacobsen et al., 2009; Mello deQueiroz et al., 2012). In contrast, FRET-based biosensors have also been applied as recombinant purified sensors to measure extracellular analytes (Bischof et al., 2017; Burgstaller et al., 2021; Namiki et al., 2007; Whitfield et al., 2015; Zhang et al., 2018). For cellular immobilization, these biosensors were further engineered using non-covalent biotin (strept/trapt) avidin interaction motifs; however, these approaches also rely on genetic manipulations of target cells or unspecific biotinylation of the cell surface (Burgstaller et al., 2021; Namiki et al., 2007; Whitfield et al., 2015; Zhang et al., 2018).

A more precise targeting specificity can be achieved by using antibodies or fragments thereof. Binding molecules derived from heavy chain only antibodies of camelids, termed VHHs or nanobodies (Nbs), have proven to be reliable tools for many applications in biomedical research, diagnostics, or even therapy (Hamers-Casterman et al., 1993; Liu et al., 2021; Muyldermans, 2021; Wagner and Rothbauer, 2021). Nbs are characterized by small size, antibody-like affinities and specificities, low off-target accumulation, high stability and good solubility (Muyldermans, 2013). Their unique properties and ease of genetic and/or chemical functionalization offer significant advantages over conventional antibodies. Recently, Nbs specific for GFP or RFP which were genetically fused with different variants of the fluorescent Ca^2+^ sensor GECO1.2 (Zhao et al., 2011) have been used to measure physiological Ca^2+^ changes in living cells after extrinsic stimulation. Similarly, the green fluorescent pH sensor super-ecliptic pHluorin (SEpHluorin) (Sankaranarayanan et al., 2000) or the red fluorescent pH sensor pHuji (Shen et al., 2014) and the excitation ratiometric ATP/ADP sensor Perceval-HR (Tantama et al., 2013) were combined with these Nbs to specifically measure pH shifts or ATP/ADP losses after inhibition of glycolysis and oxidative phosphorylation at distinct cellular compartments within live cells (Prole and Taylor, 2019).

In this study, we exploit the potential of Nbs to immobilize functional biosensors as recombinant proteins on the extracellular surface. Therefore, we developed biosensor fusion constructs using either a peptide tag-specific Nb (SPOT-Nb) (Braun et al., 2016) as a broadly applicable generic binding molecule, or a HER2-specific Nb (2Rs15d) (Vaneycken et al., 2011) targeting an endogenous surface protein in combination with GEPII 1.0 (Bischof et al., 2017), pH-Lemon (Burgstaller et al., 2019), or FLII12Pglu-700μδ6 (further referred to as FLII) (Takanaga et al., 2008) to measure extracellular K^+^, pH, and glucose changes near the cell surface. Our results showed that these sensors can be successfully immobilized on the cell surface and retain their full functionality. Most importantly, the Nb-fused biosensors enabled spatially resolved physiologically relevant FRET-based or fluorescent measurements of extracellular changes for all analytes tested over an extended period of time. From our findings, we propose that this versatile approach opens new opportunities to study important metabolic activities at the interface of cells and the ECM in advanced experimental settings, including 3D organoids or possibly *in vivo* models.

## Results

### Generation and characterization of fluorescent biosensors fused to the SPOT-Nb

**Figure 1:**
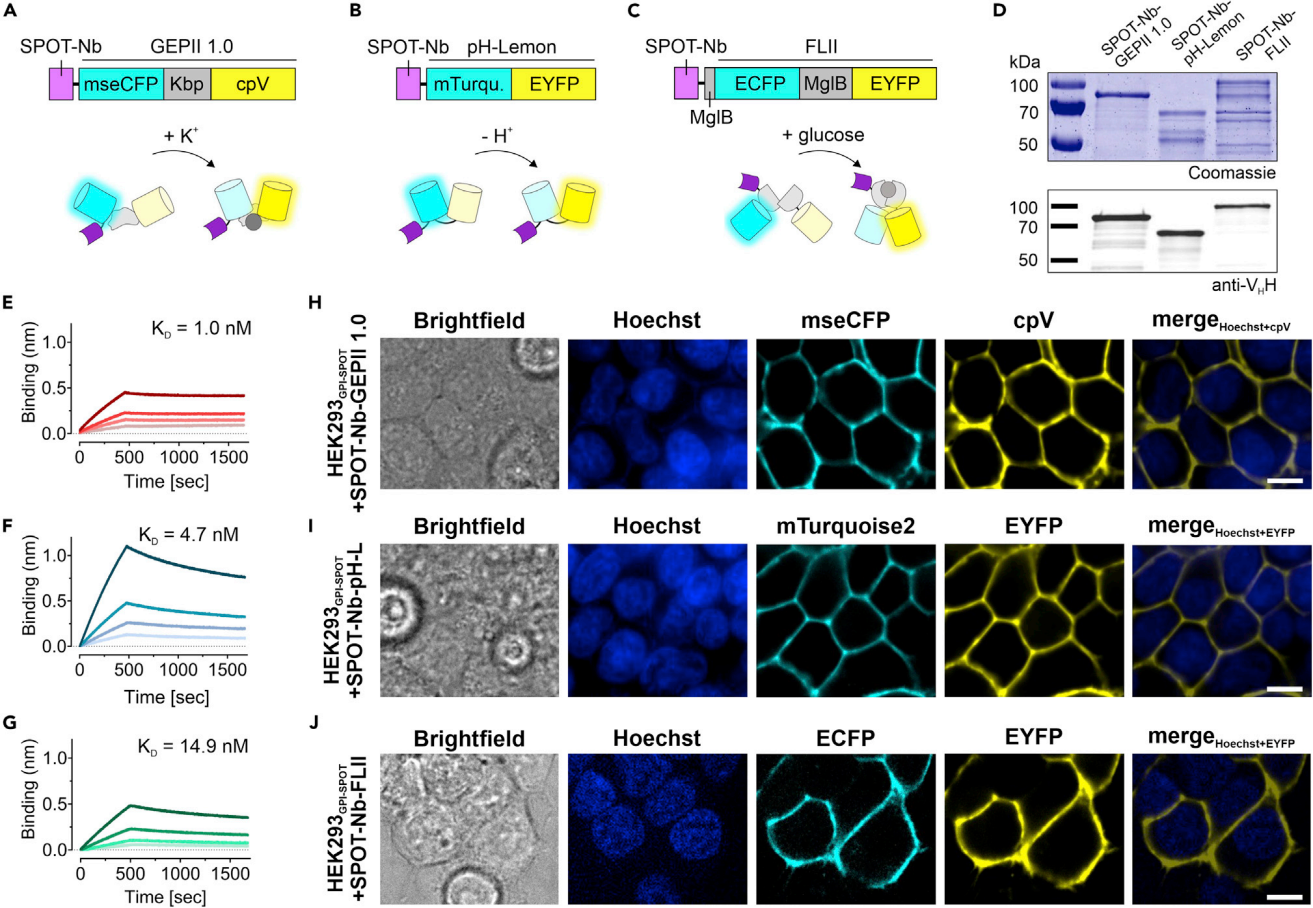
Fluorescent biosensors fused to the SPOT-Nb specifically bind the SPOT-tag Schematic illustration of (A) the SPOT-Nb-GEPII 1.0 consisting of the Nb (magenta), the FRET donor (mseCFP, cyan), the potassium binding domain (Kbp, gray) and the FRET acceptor (cpV, yellow), responding to K+ alterations by conformational rearrangement of Kbp, (B) the SPOT-Nb-pH-Lemon consisting of the Nb (magenta) fused to the pH stable mTurquoise2 (cyan), and the pH sensitive EYFP (yellow), responding to pH alterations due to quenching of the EYFP fluorescence, (C) the SPOT-Nb-FLII comprising the Nb (magenta) domain, an FRET donor (ECFP, cyan), the separated glucose binding domains (MglB, gray) and the FRET acceptor (EYFP, yellow), responding to glucose alterations by conformational rearrangement of the MglB domains. (D) Recombinant expression and purification of the Nb-fused biosensors using immobilized metal ion chromatography (IMAC) and size exclusion (SEC). Coomassie-stained SDS-PAGE of 1 μg (upper panel) and immunoblot analysis using anti-VHH antibody (lower panel) of purified SPOT-Nb-GEPII 1.0, SPOT-Nb-pH-Lemon and SPOT-Nb-FLII proteins are shown. (E–G). For biolayer interferometry (BLI)-based affinity measurements, biotinylated SPOT peptide was immobilized on streptavidin biosensors and the protein concentration of full-length SPOT-Nb fused biosensors was determined by densitometric analysis. Kinetic measurements were performed using four concentrations of purified Nb-fused biosensors ranging from 5–40 nM (SPOT-Nb-GEPII 1.0), 2.5–20 nM (SPOT-Nb-pH-Lemon) and 2.5–20 nM (SPOT-Nb-FLII). Sensograms of SPOT-Nb-GEPII 1.0 (E), SPOT-Nb-pH Lemon (F) and SPOT-Nb-FLII (G) are shown. (H–J) Representative confocal microscopy images of live HEK293 cells expressing GPI-anchored SPOT-tag (GPI-SPOT) on the plasma membrane upon incubation with SPOT-Nb-GEPII 1.0 (H); SPOT-Nb-pH Lemon (I) and SPOT-Nb-FLII (J). Shown from left to right are: Brightfield, Hoechst, and the fluorescent signals of the respective FPs as indicated. Scale bar 10 μm.

To measure changes in K^+^, pH, and glucose as important TME-associated analytes in the extracellular space, three FRET biosensors were used. GEPII 1.0 represents a highly specific indicator for K^+^, which is based on a conformational rearrangement mediated by the K^+^ binding protein Kbp (Figure 1A) (Bischof et al., 2017). pH-Lemon, a pH sensor, which is based on the intrinsic pH sensitivity and insensitivity of EYFP and mTurquoise2, respectively (Figure 1B) (Burgstaller et al., 2019), and FLII, a widely used glucose indicator, which permits glucose sensing by conformational changes of the glucose binding domain, MglB, thereby increasing FRET (Figure 1C) (Takanaga et al., 2008). To generate Nb-fused biosensors, the SPOT-Nb was fused N-terminally to the different FRET pairs (Figures 1A–C) (Braun et al., 2016). This well-established Nb binds the SPOT peptide (SPOT-tag), with high specificity and affinity and provides optimal properties for validating the applicability of Nb-mediated immobilization of biosensors on the cell surface. As a first step, all Nb-fused biosensors were cloned with a C-terminal His_6_-tag, expressed in *Escherichia coli* (*E.coli*) and purified using immobilized metal ion affinity chromatography (IMAC) followed by size exclusion chromatography (SEC). SDS-PAGE analysis from the last purification step showed that all three sensor constructs are expressed and yielded at the expected molecular weight as full-length sensor fusion protein of SPOT-Nb-GEPII 1.0 (86 kDa), SPOT-Nb-pH-Lemon (71 kDa) and SPOT-Nb-FLII (103 kDa) (Figure 1D). However, for SPOT-Nb-pH-Lemon and SPOT-Nb-FLII additional bands referring to smaller proteins were detected. Immunoblot analysis using an anti-V_H_H antibody revealed that the smaller proteins were lacking the SPOT-Nb moiety (Figure 1D). These findings indicated that the chimeric SPOT-Nb-pH-Lemon and SPOT-Nb-FLII constructs are sensitive to degradation that occurs throughout the expression or purification process. However, since the resulting protein fragments could not bind the SPOT-tag, we concluded that they should not interfere with subsequent measurements based on Nb binding and sensor functionality. Next, we investigated whether the SPOT-Nb (∼15 kDa) fused to the large sensor proteins (∼52–88 kDa) exhibits still full binding capacity. To this end, we first quantified the amount of full-length SPOT-Nb fused biosensors in approximation by densitometric analysis of Coomassie-stained gels (Figure 1D) followed by determining their binding affinities to the isolated SPOT-peptide using biolayer interferometry. Strong binding of the Nb-fused biosensors to the SPOT-tag with dissociation rate constants (K_D_) in the low nanomolar range of ∼1 and ∼4.7 nM for SPOT-Nb-GEPII 1.0 (Figure 1E and Table S1) and SPOT-Nb-pH-Lemon (Figure 1F and Table S1), respectively were determined. These affinities are comparable to those previously measured for the SPOT-Nb alone (Braun et al., 2016). However, a slightly higher K_D_ of ∼14.8 nM was determined for the SPOT-Nb-FLII (Figure 1G and Table S1) which could be because of a steric hindrance caused by the large biosensor moiety of ∼88 kDa.

### SPOT-Nb-biosensors bind the SPOT-tag on the surface of living cells

To test the binding properties in a more relevant setting, we examined the ability of the purified SPOT-Nb-fused biosensors to bind to GPI-anchored SPOT tags (GPI-SPOT) on the surface of HEK293 cells. Therefore, HEK293 either transiently transfected with a GPI-SPOT expression construct or left untreated (HEK293 wildtype (WT)) were incubated with the SPOT-Nb-biosensor fusion constructs (Figures 1H–J and S1A), or a fluorescently labeled SPOT-Nb (SPOT-Nb_ATTO488_) with a small ATTO488 fluorophore (∼1 kDa) as a positive control (Figure S1B). Live-cell fluorescent imaging resulted in strong fluorescence signals exclusively localized at the plasma membrane of HEK293 cells expressing the GPI-SPOT (Figures 1H–J). The obtained signal intensities and localization were comparable to the respective SPOT-Nb_ATTO488_ staining (Figure S1B). In contrast, no fluorescence signals were detected on non-transfected HEK293 cells (Figure S1A) or upon incubation of cells expressing GPI-SPOT with recombinant purified GEPII 1.0 lacking the SPOT-Nb (Figure S1C). From these findings, we concluded that fusion of the SPOT-Nb to the sensors did not affect the binding properties of the Nb and that all tested SPOT-Nb-fused biosensors are suitable to identify cells presenting the SPOT-tag on the extracellular surface of their plasma membrane.

Next, we investigated the stability of binding and potential internalization of the SPOT-Nb biosensors over time, as both could limit the reliable and sustained measurement of extracellular analytes. Thus, GPI-SPOT expressing HEK293 cells were incubated with SPOT-Nb-GEPII 1.0 and SPOT-Nb-pH-Lemon and time-lapse imaging of the fluorescence signals of the biosensors was performed for 4.5 h. For both Nb-fused biosensors, the corresponding fluorescent images showed that signals were retained specifically at the plasma membrane. Endocytic vesicles containing fluorescence signals were rarely observed (Figures S2A and S2B). To follow these processes for an even longer period, we continued and additionally re-investigated the cells after 48 h of initial immobilization. We observed that all tested Nb-fused biosensors were nearly undetectable 48 h after immobilization, possibly due to disassociation or degradation. However, reloading with SPOT-Nb-GEPII 1.0 resulted in a similar membrane staining pattern compared to earlier time points (Figure S3). Overall, these results showed that the biosensor constructs fused with SPOT-Nb are capable of transiently and specifically targeting the plasma membrane over an extended period of time. We further concluded that these sensors can be used for long-term experiments, although cell re-staining may be required.

### SPOT-Nb-biosensors detect changes in extracellular analytes

**Figure 2:**
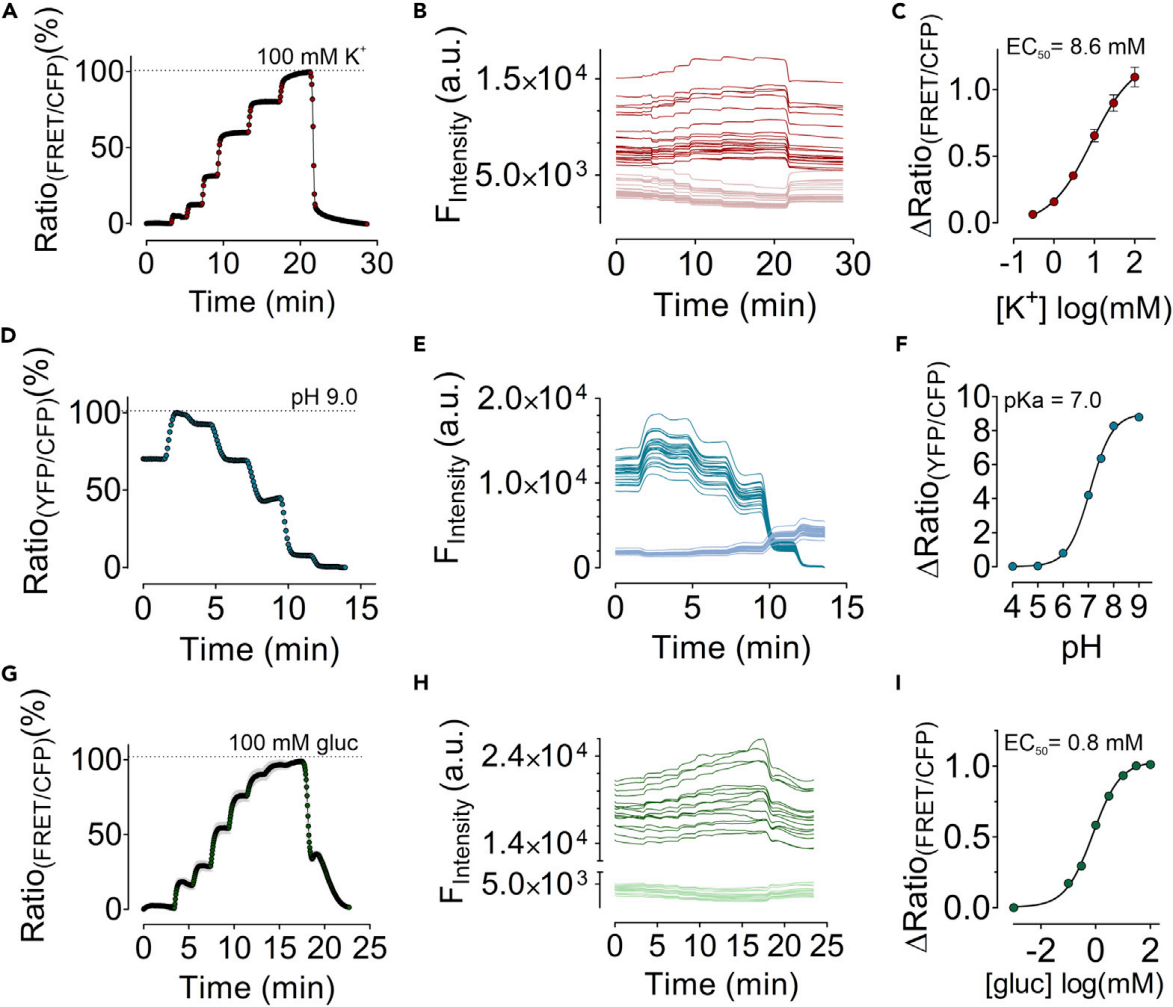
Biosensors immobilized on the plasma membrane respond to K^+^, pH and glucose alterations (A) Response of SPOT-Nb-GEPII 1.0 immobilized on HEK293 cells expressing GPI-SPOT upon perfusion of buffers with different [K^+^] over time. Shown is the mean ± SD of one representative measurement of multiple cells (n= 23). (B) Single cell traces of cpV (red) and mseCFP (pink) of immobilized SPOT-Nb-GEPII 1.0 in response to buffers with different [K+] as shown in panel (A). (C) Dose-response curve of SPOT-Nb-GEPII 1.0 with an EC50 of 8.6 mM (4.8–15.5 mM). Shown is the mean ± SEM of four biological replicates including 50 cells in total. (D) Response of SPOT-Nb-pH-Lemon immobilized on HEK293 cells expressing GPI-SPOT upon perfusion of buffers with different pH over time. Shown is the mean ± SD of one representative measurement of multiple cells (n= 25). (E) Single cell traces of mTurquoise2 (blue) and EYFP (petrol) of immobilized SPOT-Nb-pH-Lemon in response to buffers with different pH as shown in panel (D). (F) Dose-response curve of SPOT-Nb-pH-Lemon with a pKa of 7.0 (7.0–7.1) Shown is the mean ± SEM of three biological replicates including 52 cells in total. (G) Ratiometric response of SPOT-Nb-FLII immobilized on HEK293 cells expressing GPI-SPOT upon perfusion of buffers with different glucose levels over time. Shown is the mean ± SD of one representative measurement of multiple cells (n= 18). (H) Single cell traces of ECFP (light green) and EYFP (dark green) immobilized SPOT-Nb-FLII in response to buffers with different pH as shown in panel (G). (I) Dose-response curve of SPOT-Nb- FLII with an EC_50_ of 0.8 mM (0.6–1 mM). Shown is the mean ± SEM of three biological replicates including 44 cells in total.

So far, our results have shown that the biosensors fused with SPOT-Nb can specifically recognize the antigen expressed on the surface of living cells. Hence, the next question was whether the immobilized biosensors are capable of dynamically detecting corresponding changes in extracellular analytes with an appropriate signal-to-noise ratio and sensitivity. First, we examined the fluorescence emission signals of mseCFP and FRET of SPOT-Nb-GEPII 1.0 interacting with GPI-SPOT at the surface of HEK293 cells in response to the administration of buffers with different K^+^ concentrations ([K^+^]). Before the analysis, we confirmed the correct localization of the Nb-fused biosensor by fluorescent live-cell imaging revealing specific staining of the plasma membrane (Figures S4A and S4B). Subsequently, the cells were perfused with buffers comprising increasing [K^+^] ranging from 0–100 mM, and FRET signals were continuously visualized (Figure 2A and Video S1). In line with the [K^+^]-dependent FRET signals obtained (Figure 2A), the fluorescence emission signals of the single FPs displayed a ratiometric behavior with decreasing mseCFP and increasing FRET fluorescence emissions (Figure 2B). To visualize the dynamics of the sensor response, FRET ratio movies were created from images of time-lapse series showing analyte changes as a pseudo-colored code (Video S1). The immobilized SPOT-Nb-GFP1 1.0 responded dynamically to K^+^ changes in the extracellular compartment and displayed concentration-dependent FRET ratio signals (Video S1). The calculated half-maximal concentration (EC_50_) of 8.6 mM (Figure 2C), indicated that SPOT-Nb-GEPII 1.0 covers a (patho-) physiologically relevant range of extracellular [K^+^] ([K^+^_ex_]) (Eil et al., 2016). Next, we investigated the sensitivity of the immobilized SPOT-Nb-pH Lemon. For this purpose, cells were exposed to different extracellular pH values (pH 5–9). Upon separate excitation of mTurquoise2 and EYFP, as previously reported (Burgstaller et al., 2019), SPOT-Nb-pH Lemon showed ratiometric changes dependent on extracellular pH (pH_ex_) (Figures 2D and S4C). Dynamic measurements revealed an instant and homogeneous decrease in the FRET ratio signals in response to extracellular acidification, as indicated by the color switch from white/red to blue (Video S2). Based on the sensor design, SPOT-Nb-pH-Lemon repeatedly responded to decreasing pH_ex_ with increasing mTurquoise2 fluorescence because of the pH stability of FP, which as an FRET donor, however, is affected by the attached pH-sensitive EYFP. As expected, decreasing EYFP fluorescence could be observed upon acidification, caused by the protonation of the fluorophore and, subsequently, quenching of the FP (Figures 2E and S4D). Considering the calculated pKa value of 7.0 (Figure 2F), which corresponds well with the previously reported pH-sensitivity of the sensor (Burgstaller et al., 2019, 2021), we concluded that immobilized SPOT-Nb-pH Lemon is suitable to detect physiologically relevant changes in pH_ex_ (Gallagher et al., 2008). In contrast to Nb-fused GEPII 1.0 and pH-Lemon, the FLII biosensor exhibits a more complex biosensor design as it relies on the formation of a functional glucose binding domain based on two split MglB fragments (Deuschle et al., 2005; Takanaga et al., 2008). However, as we inserted a long flexible linker between the Nb and the first MglB fragment we hypothesized that this design would confer sufficient steric flexibility for glucose binding and FRET signal generation. Hence, the functionality of SPOT-Nb-FLII to detect alterations of the extracellular glucose concentration ([GLU]_ex_) was tested upon cellular immobilization on HEK293 GPI-SPOT cells (Figures S4E and S4F). Cells were perfused with buffers comprising increasing [Glu]_ex_ ranging from 0–100 mM (Figure 2G). Similar to the SPOT-Nb-GEPI 1.0 and SPOT-Nb-pH Lemon, the plasma membrane-bound SPOT-Nb-FLII biosensor dynamically displayed changes in extracellular glucose levels over time (Video S3). Continuous measurement of the FRET ratio signals revealed a distinct, ratiometric response of the immobilized Nb-fused biosensor to [GLU]_ex_ alterations in the low mM range (Figures 2G and 2H) with an estimated EC_50_ of 0.9 mM (Figure 2I). These findings demonstrated that all SPOT-Nb fused biosensors retain their functionality and report changes in extracellular analytes and conditions in a physiologically relevant range. Apparently, the N-terminal Nb-binding moiety does not negatively affect functional biosensor conformation, which may be because of the presence of a long flexible linker between the two functional domains.

Video S1. Visualization of K^+^ alterations on the plasma membrane using immobilized SPOT-Nb-GEPII 1.0 displayed as pseudo-colored FRET ratios on HEK293 cells expressing GPI-SPOT, related to Figure 2A The FRET ratio video was generated using fluorescence widefield microscopy over time upon perfusion of buffers with different [K^+^]. The pseudo-colored FRET ratio video was generated by dividing the acquired image stacks of the FRET and CFP fluorescence emission (FRET/CFP) using ImageJ. FRET ratio scale was set accordingly to display minimal FRET ratios in blue (low K^+^) and maximal FRET ratios in red/white (high K^+^). Buffer exchanges are shown within the video.

Video S2. Visualization of pH alterations on the plasma membrane using immobilized SPOT-Nb-pH-Lemon displayed as pseudo-colored FRET ratios on HEK293 cells expressing GPI-SPOT, related to Figure 2D The pseudo-colored FRET ratio video was generated using fluorescence widefield microscopy over time upon perfusion of buffers with different pH. The FRET ratio video was generated using ImageJ by dividing the acquired image stacks of the CFP and YFP fluorescence emission (CFP/YFP). FRET ratio scale was set to display minimal FRET ratios in blue (alkaline pH) and maximal FRET ratios in red/white (acidic pH). Buffer exchanges are displayed within the video.

Video S3. Visualization of glucose alterations on the plasma membrane using immobilized SPOT-Nb-FLII displayed as pseudo-colored FRET ratios on HEK293 cells expressing GPI-SPOT, related to Figure 2G The pseudo-colored FRET ratio video was generated using fluorescence widefield microscopy over time upon perfusion of buffers with different [glucose]. The FRET ratio video was generated by dividing the acquired image stacks of the FRET and CFP fluorescence emission (FRET/CFP) using ImageJ. FRET ratio scale was set to display minimal FRET ratios in blue (low glucose) and maximal FRET ratios in red/white (high glucose). Buffer exchanges are displayed within the video.

### SPOT-Nb-GEPII visualizes K^+^ efflux from neurons

**Figure 3:**
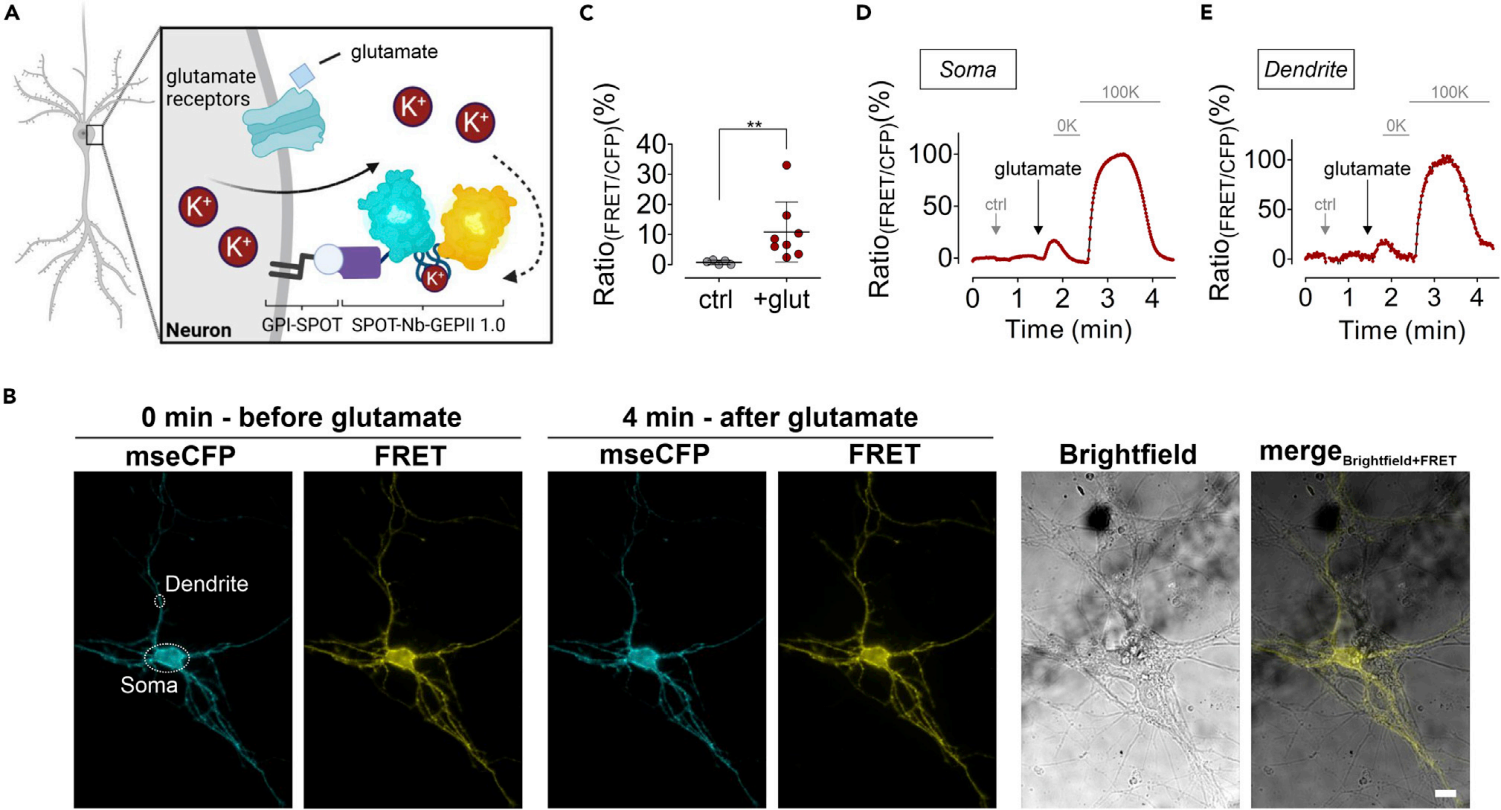
K^+^ sensors immobilized using SPOT-Nb can be used to track neuronal, glutamate-induced K^+^ efflux (A) Schematic illustration of glutamate-induced K^+^ efflux from primary hippocampal mouse neurons. Glutamate (light blue) binding to glutamatergic receptors (glutamate receptors, light green) located in the plasma membrane causes K^+^ efflux, which can be monitored by SPOT-Nb-GEPII 1.0 immobilized on the surface of primary hippocampal mouse neurons expressing GPI-SPOT. Figure created using BioRender. (B) mseCFP and FRET widefield images of SPOT-Nb-GEPII 1.0 immobilized on the membrane of a primary hippocampal neuron before (0 min) and after (4 min) addition of glutamate, washing and re-addition of K^+^, as well as brightfield and a merging of brightfield and FRET are shown. White circles indicate measurement region as shown in (D) and (E) at the soma and dendrites. Scale bar 10 μm. (C) Average (black lines) and single cell ratio changes in response to addition of a vehicle (ctrl, gray dots) and glutamate (red dots). Mann–Whitney test, p = 0.0016. (D and E) Ratiometric response of SPOT-Nb-GEPII 1.0 upon injection of a vehicle control (first arrow, ctrl), injection of glutamate (second arrow) and upon perfusion with K^+^-free buffer (0K) and subsequently with buffer containing 100 mM K^+^ (100K) measured at the soma (D) or at the dendrite (E) as indicated in (B).

Considering that we have so far evaluated Nb-fused biosensors exclusively for the detection of externally induced [K^+^]_ex_, pH_ex_ and [GLU]_ex_ alterations, we next aimed to investigate the suitability of our approach for monitoring endogenously elicited signals. To demonstrate the relevance and feasibility of our approach, we analyzed the performance of the SPOT-Nb-GEPII 1.0 to detect changes in [K^+^]_ex_ using primary hippocampal mouse neurons. Neurons transiently expressing the GPI-SPOT construct were incubated with glutamate that massively increases [K^+^]_ex_ (Figure 3A) (Burgstaller et al., 2021; Ehinger et al., 2021; Hösli et al., 1981). Immobilization of the SPOT-Nb-GEPII on primary hippocampal mouse neurons was verified by live-cell imaging as described (Figure 3B). In the following, we performed real time FRET analysis and monitored glutamate-mediated release of endogenous K^+^ to the extracellular space. First, we added pure buffer, followed by treating the cells with a glutamate bolus. Subsequently, a perfusion-mediated K^+^ wash out was performed, followed by the addition of 100 mM [K^+^]_ex_. The FRET ratio signals of SPOT-Nb-GEPII 1.0 remained virtually unaffected upon the addition of pure buffer, whereas injection of glutamate immediately increased FRET ratio signals, indicating glutamate-triggered K^+^ efflux from individual neurons (Figures 3C and 3E). Interestingly, the recorded FRET signals did not differ significantly under these conditions, either at the soma (Figure 3D) or at the dendrites (Figure 3E). In addition, dynamic measurements showed homogeneous membrane-derived FRET ratio signals with an increase in FRET ratio, i.e., an increase in white/red coloration in response to glutamate (Video S4). These results suggest that K^+^ is either evenly released from different cellular compartments or that the changes in [K^+^]_ex_ are subsequently detected by sensor molecules immobilized on different parts of the neurons. To ensure sensor functionality and classify the glutamate-induced FRET signals, FRET generation and decline were assessed in response to a K^+^-free or a buffer containing 100 mM K^+^ (Figures 3D and E). Importantly, our observations are in line with the previously reported decline of intracellular FRET [K^+^]_i_ signals monitored by GEPII 1.0 in glutamate exposed primary cerebellar granule cells (Ehinger et al., 2021). Hence, this proof-of-concept study indicates that the SPOT-Nb-GEPII 1.0 is also functional to report and monitor (patho-) physiological K^+^ efflux from neurons.

Video S4. Visualization of glutamate induced, neuronal K^+^ efflux using immobilized SPOT-Nb-GEPII 1.0 displayed as pseudo-colored FRET ratios on primary hippocampal mouse neurons expressing GPI-SPOT, related to Figure 3 The FRET ratio video was generated using fluorescence widefield microscopy and by dividing the acquired image stacks of the FRET and CFP fluorescence emission (FRET/CFP) using ImageJ. Shown are the FRET ratios over time at 0 mM K^+^ (=basal), followed by addition of a glutamate bolus (+glutamate). Subsequently glutamate was washed-out by perfusion with 0 mM K^+^, followed by 100 mM K^+^, representing the maximal FRET response. Treatment and buffer exchanges are displayed within the video.

### HER2-Nb-fused biosensors monitor [K^+^]_ex_ changes upon immobilization on HER2 expressing HEK293 cells

**Figure 4:**
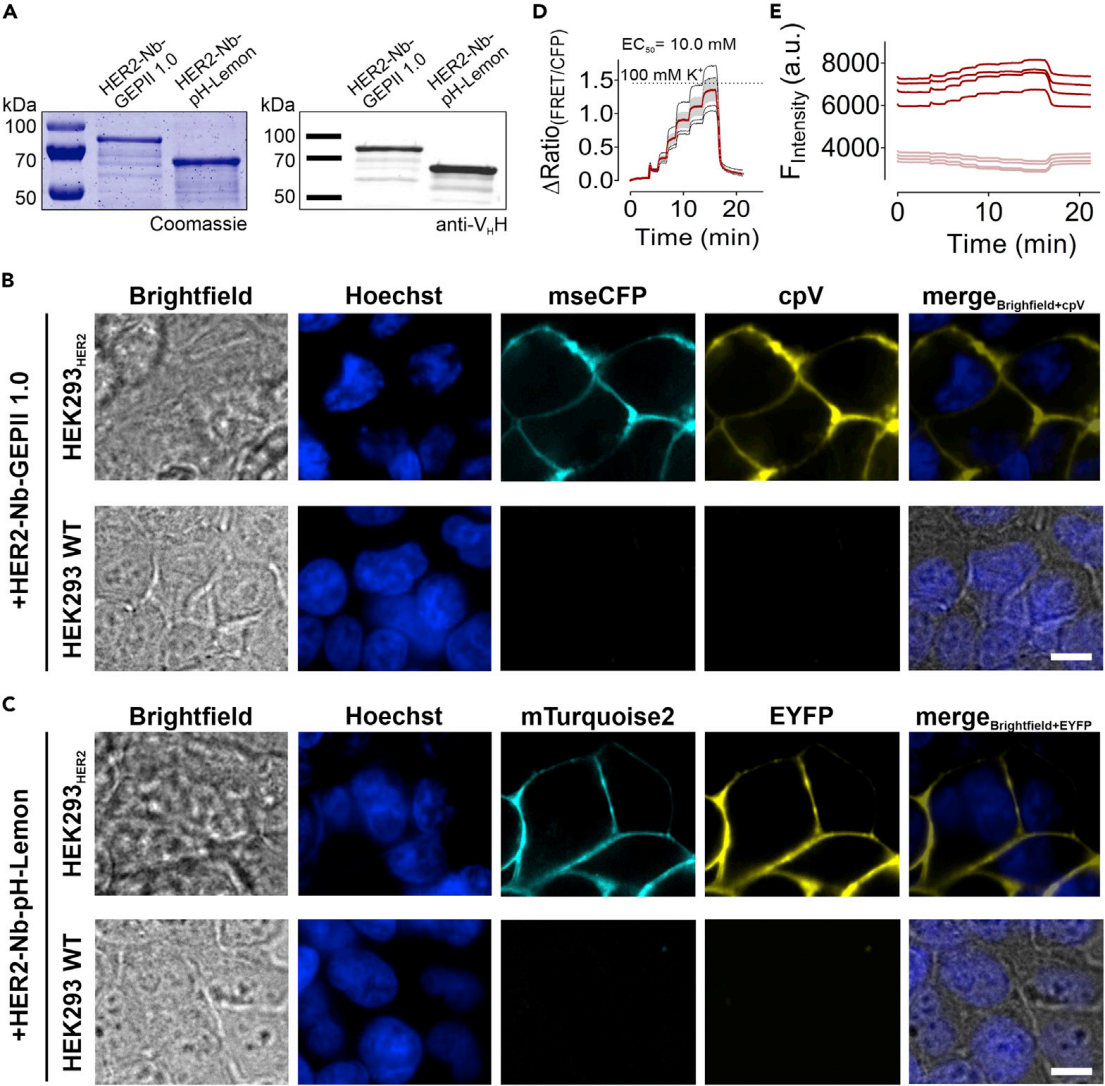
Biosensors fused to HER2-Nb precisely report [K^+^]_ex_ changes upon immobilization on HER2 expressing HEK293 cells (A) Coomassie-stained SDS PAGE of 1 μg protein (left panel) and immunoblot analysis using anti-VHH antibody (right panel) of purified HER2-Nb-GEPII 1.0 and HER2-Nb-pH-Lemon proteins are shown. (B and C) Representative confocal images of living HEK293 cells transiently overexpressing HER2 (upper row) or untransfected HEK293 cells (lower row) following incubation with HER2-Nb-GEPII 1.0 (B) or HER2-Nb-pH-Lemon (C) are shown. Scale bar 10 μm, N= 4. (D) Response of HER2-Nb-GEPII 1.0 immobilized on HEK293 cell transiently overexpressing HER2 in response to buffers with different K^+^. Shown is a representative measurement of four cells (mean ± SD in red, traces from individual cells in black). (E) Respective single wavelength traces (FRET in red, mseCFP in pink) of the ratio curve as shown in (D) in response to K^+^ alterations.

Notably, all these results were obtained with SPOT-Nb fused biosensors, which require the expression of the SPOT-tag as a broadly applicable but artificial antigen on the plasma membrane. To further analyze whether this approach can also be used for endogenous cell surface epitopes, we selected a Nb (2Rs15d), which has been previously described to bind human epidermal growth factor receptor 2 (HER2) - a plasma membrane receptor widely expressed in breast cancer (Vaneycken et al., 2011). Following our original strategy, we designed and generated HER2-Nb-fused GEPII 1.0 or pH-Lemon biosensors in analogy to SPOT-Nb-based constructs. Bacterial expression yielded intact recombinant proteins showing only minor degradation as demonstrated by SDS-PAGE and immunoblotting (Figure 4A). We further performed live-cell imaging analysis on HEK293 cells transiently expressing human HER2. Cells staining with purified HER2-Nb-GEPII 1.0 (Figure 4B) and HER2-Nb-pH-Lemon (Figure 4C) resulted in prominent staining of the plasma membrane, whereas untransfected HEK293 cells remained unstained (Figures 4B and C). In addition, incubation of HEK293 cells expressing HER2 with GEPII 1.0 lacking the Nb also showed no fluorescence signal (Figure S5). The imaging results indicated that both HER2-Nb fused biosensors can specifically recognize and properly bind their cell-surface target.

To elucidate the functionality of the biosensors, we tested HER2-Nb-GEPII 1.0, as its functional principle is more complex because of the conformational change, compared to the fluorescence quenching of pH-Lemon. Perfusion of HER2 expressing HEK293 with increasing [K^+^]_ex_ (0–100 mM) revealed the functionality of HER2-Nb-GEPII 1.0 construct for detecting alterations in the [K^+^]_ex_ (Figure 4D). Similarly, HER2 Nb-fused GEPII 1.0 exhibited ratiometric behavior with increasing FRET and decreasing mseCFP fluorescence in response to increasing [K^+^]_ex_, revealing an EC_50_ value for K^+^ of 10 mM (Figures 4D and E) which is well in the range of SPOT-Nb-GEPII 1.0 (Figure 2C).

### HER2-Nb-biosensors specifically label endogenous HER2 on breast cancer cells

**Figure 5:**
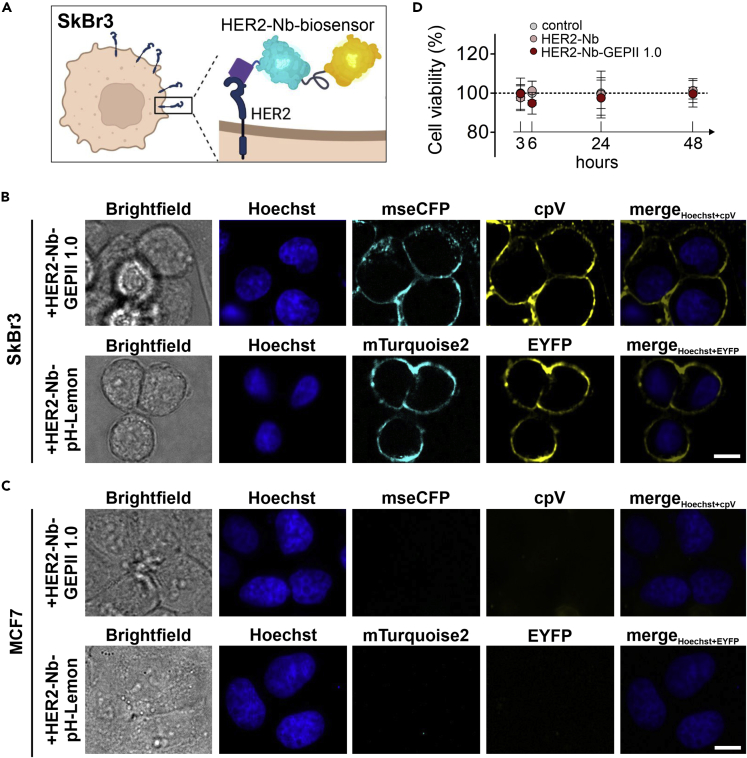
HER2-Nb-biosensors specifically label endogenous HER2 on HER2 positive breast cancer cells (A) Schematic illustration of a HER2 positive SkBr3 cell endogenously expressing HER2 on the cell surface. Biosensors fused to HER2 can be bound to HER2 for immobilization on the plasma membrane. Figure created using BioRender. (B) Representative images of SkBr3 cells following incubation with HER2-Nb-GEPII 1.0 and HER2-Nb-pH-Lemon. Scale bar 10 μm, n= 4 experiments representing biological replicates. (C) Representative confocal images of HER2 negative MCF7 breast cancer cells following incubation with HER2-Nb-GEPII 1.0 and HER2-Nb-pH-Lemon are shown. Scale bar 10 μm, N= 4. (D) Determination of cell viability of SkBr3 cells using MTT in response to vehicle (ctrl), unfused HER2-Nb and HER2-Nb-GEPII 1.0 after 3, 6, 24, and 48 h after immobilization. Not significant as determined using one-way ANOVA, Dunn’s Multiple Comparison Test.

Having validated their functionality, we finally tested whether the recombinant HER2-Nb-fused biosensors can be immobilized on cells endogenously expressing HER2. Therefore, we utilized two breast cancer cell lines either positive (SkBr3) or negative (MCF7) for human HER2 (Figure 5A). Upon incubation with both Nb-fused biosensors, live-cell imaging showed a clear fluorescence staining of the cell surface for HER2-Nb-GEPII 1.0 and HER2-Nb-pH-Lemon for SkBr3 cells (Figure 5B) but not for MCF7 cells (Figure 5C). The HER2-Nb mediated specificity was further confirmed by the absence of any fluorescent staining of SkBr3 and MCF-7 cells upon incubation with GEPII 1.0 lacking the HER2-Nb (Figure S5). Additional time-lapse imaging showed a stable signal at the plasma membrane for 4.5 h, despite some endocytosed vesicles (Figures S6A and S6B), which may indicate that cell viability is not affected by immobilization of the biosensors via the HER2-Nb. In line with these findings, an MTT-based assay showed that labeling of SkBr3 cells with HER2-Nb or HER2-Nb-GEPII 1.0 did not affect cell viabilities compared with control cells at 3, 6, 24, and 48 h (Figure 5D).

To further test the applicability of HER2-Nb-GEPII 1.0 for measuring physiological K^+^ release from dying tumor cells, we simulated necrotic cell death by applying digitonin to permeabilize the cells, resulting in K^+^ release (Figures S7A and S7B). [K^+^_ex_] increased shortly after treatment with digitonin, which was accompanied by morphological changes such as cell swelling, membrane rupturing (Figure S7C) and, subsequently, cell death (Figure S7D and Video S5). Based on these observations, we assume that due to their stable immobilization in combination with low cell toxicity, recombinant Nb-fused biosensors might also be applied for more complex models such as 3D cell models or patient-derived organoids.

Video S5. Visualization of digitonin-induced cell death of SkBr3 cells, related to Figure S7 SkBr3 cells were imaged using widefield microscopy with image acquisition every 10 s. At the time point indicated in the video, 50 μL of DMSO (diluted in buffer according to digitonin) or 50 μL of a 200 μM digitonin solution were pipetted onto the cells to determine digitonin-induced morphological changes, e.g., cell membrane rupturing and cell death.

## Discussion

Here, we provide evidence that recombinant Nb-fused FRET biosensors enable the visualization of dynamic analyte alterations in the extracellular compartment. Changes in ions and metabolites were visualized in a single-cell resolution and in real-time. Such approaches are relevant for optical mapping of pathophysiological relevant alterations of discrete analytes in extracellular fluids i.e., in the vasculature, lymphatic vessels, body cavities, synovial compartments, the cerebrospinal fluid as well as the TME. Moreover, the flexible combination of various, target-specific Nbs with different sensor molecules makes our toolbox approach versatile applicable.

The obtained results indicate that both heterologously expressed and endogenous plasma membrane epitopes are specifically targeted with different nanobodies such as SPOT-Nb (Braun et al., 2016) and HER2-Nb (Vaneycken et al., 2011). Of importance, immobilization of the Nb-fused FRET biosensors on their target side does not interfere with the conformational rearrangement as shown for the potassium sensor GEPII 1.0 (Bischof et al., 2017) and the glucose sensor FLII12Pglu700μd6 (Takanaga et al., 2008), nor the extinction of YFP fluorescence at low pH as shown for pH-Lemon (Burgstaller et al., 2019), which are necessary to produce accurate and reliable FRET signals, relying on different sensor principles. Although different in the mode of action, biosensors immobilized on the cell surface expressing the Nb targets remained fully functional for visualizing physiologically relevant changes in K^+^, pH, and glucose (Eil et al., 2016; Gallagher et al., 2008; Takanaga et al., 2008). The respective EC_50_ values obtained upon Nb immobilization were comparable to previously reported values (Burgstaller et al., 2021; Takanaga et al., 2008). Vice versa, the fusion of quite bulky biosensors to the much smaller Nb did not alter the affinity of the Nb. Accordingly, Nb-fused FRET biosensors displayed similar affinities in the low nanomolar range despite their significant increase in molecular weight compared to the unfused Nb (Braun et al., 2016; Vaneycken et al., 2011). Notably, for the generation of the Nb-fused biosensors, we chose a flexible design with long linker sequences that allow maximum steric freedom of the functional subunits. Although all Nb-fused biosensors could be functionally expressed in bacterial systems, they differed in their protein integrity and stability, which could be due to the different requirements needed for the correct formation of each functional subunit. In particular, the proper formation of essential disulfide bonds within the immunoglobulin fold of a Nb requires the oxidative environment of the bacterial intermembrane space, whereas FPs such as CFP or YFP are preferentially expressed in the reducing environment of the cytoplasm (Feilmeier et al., 2000; Jain et al., 2001; Kunz et al., 2018). To improve expression conditions in the future, additional expression of chaperones such as disulfide isomerase (DsbC) should be considered (Olichon and Surrey, 2007).

By now, multiple FRET-based approaches were developed to monitor analytes in a single cell compartment or within the entire cells and thus are introduced as genetically encoded constructs (Depaoli et al., 2019). However, genetic manipulation of sensor-targeted cells bears the risk of low transfection efficiencies and/or metabolic alterations that might interfere with the readout of interest (Fiszer-Kierzkowska et al., 2011; Mello deQueiroz et al., 2012). Various strategies have already been described for the specific targeting of biosensors as recombinant proteins, ranging from the well-known biotin-avidin interaction (Burgstaller et al., 2022; Zhang et al., 2018) to pH (low) insertion peptides (pHLIPs) (Weerakkody et al., 2013), which are integrated into the plasma membrane in response to low pH. Because of the acidic pH within the TME, this technique might be suitable to address this compartment in general. However, pHLIPs cannot be used to target specific proteins e.g., on cancer cells. In contrast, by using Nbs as the smallest intact antigen-binding fragments specific for cell surface markers, our approach focuses on their application to the specific study of extracellular signal mediators.

The Nb-fused GEPII 1.0 sensor presented here could readily be used to study the effects of discrete plasma membrane K^+^ channels that facilitate the flux of K^+^ ions between the cytosol and the extracellular space. Such channels show cancer specific expression patterns and are present in a variety of solid tumors (Mohr et al., 2019b; Pardo and Stühmer, 2014; Steudel et al., 2017). Notably, recently agents modulating these channels have demonstrated anti-tumor efficacy (Mohr et al., 2019a, 2020; Peruzzo and Szabo, 2019). Accordingly, our approach of a cell-specific immobilization of K^+^ biosensors might facilitate the screening and identification of such channel modulating agents. In another aspect, a recent analysis of genetically modified mouse models lacking, for instance, distinct Ca^2+^-activated K^+^ channels (KCa) revealed that this family of K^+^ channels is crucially involved in breast cancer development and its response to therapy (Mohr et al., 2019a; Steudel et al., 2017). However, the contribution of cancer-associated K^+^ channels to the TME and how their activity relates to functionally relevant changes in [K^+^]_ex_ is poorly understood (Burgstaller et al., 2022). Therefore, we anticipate that further development of our HER2-Nb-GEPII 1.0 for 3D cell models or *in vivo* applications in these models will lead to a more comprehensive understanding of the impact of this class of “onco-channels” (Huber, 2013). Previously, several studies suggested that the ionic composition and thus [H^+^]_ex_ and [K^+^]_ex_ play a role in the stromal cell recruitment at primary and metastatic tumor sites and in anti-tumor immunity (Comes et al., 2015; Huang et al., 2016; Som et al., 2016). By using combinations of the Nb-fused FRET biosensors developed here, the interplay of extracellular ions, metabolites, and conditions in response to various stimuli, such as cancer treatment, could be studied more comprehensively, thus contributing to the understanding of how extracellular conditions correlate with cancer cell proliferation and/or migration. The possibility to combine two or more Nbs with different biosensors might even broaden their application in multi-parametric imaging approaches, a technique highly desired in fluorescence microscopy (Carlson and Campbell, 2009). Finally, the biosensors proposed here can be further extended to monitor the effects of other factors such as cytokines, growth factors, hormones, ions, and metabolites that are known to determine how cells respond to their environment, mediating cell-to-cell and cell-to-matrix communication (Yang et al., 2019).

By flexibly linking two functionally independent modules, the herein presented principle of Nb-fused biosensors can be transferred to a range of signaling mediators or events utilizing appropriate FRET chromophores and Nbs that display suitable affinity and specificity for their target epitopes. In particular, Nbs against a surface marker of solid tumor cells such as epidermal growth factor receptor (EGFR) (Roovers et al., 2007), prostate-specific membrane antigen (PSMA) (Evazalipour et al., 2014), carcinoembryonic antigen (CEA) (Cortez-Retamozo et al., 2004) or directly targeting components of the ECM such as the EIIIB domain of fibronectin (Jailkhani et al., 2019) may be promising candidates for the design of additional Nb-fused biosensors in the future.

In summary, the design and application of Nb-fused biosensors as demonstrated in this study address the growing need for reliable reagents to accurately determine analytes and their changes close to cell surfaces in the extracellular space. We assume that these new tools could pave the way to a better understanding of the cell-to-cell and cell-to-matrix communication elicited by ions, metabolites, and other signaling factors. Such versatile probes will open up new possibilities for the reliable investigation of extracellular analytes.

### Limitations of the study

The approach as presented here focuses on the targeted redirection of recombinant Nb-fused biosensors to the surface of living cells to visualize changes in analytes near the plasma membrane. Using SPOT and HER2-Nb fused to GEPI 1.0, pH-Lemon, and FLII, we demonstrate feasibility for different exemplary probes, from which successful transfer to other constructs can be assumed. Nevertheless, additional aspects have to be considered. As shown, some Nb-fused biosensors suffer from reduced protein integrities following bacterial expression and purification. Although this did not interfere with immobilization and live-cell imaging, similar problems are conceivable for other constructs and may need to be addressed by alternative expression strategies and systems. In addition, the biosensors described herein were used to determine and visualize relative rather than absolute analyte changes. However, a quantitative determination would be feasible upon calibration of the biosensors (i.e., by exposure to minimal and maximal analyte concentrations included within the measurements). Most importantly, this study encompasses 2D cell culture models what limits the informative value with regard to the analysis of physiological analyses or within, for instance, the TME. Also, substantial differences in response to certain treatments due to the usage of freshly isolated cells (e.g., primary neurons), which might differ in their metabolism has to be considered.

As a next step, we aim to use the Nb-fused biosensors in 3D cell culture models. In such models the extracellular fluid between individual cells is significantly reduced, which favors the detection of small analyte changes. Notably, such applications need an in-depth characterization because the size of the Nb-biosensor constructs (ranging from 70 kDa for Nb-pH-Lemon to 103 kDa for Nb-FLII) might represent a limiting step for optimal distribution and diffusion of the probes to all potential target sites within a 3D tissue culture. However, increasing the incubation time or changing the temperature during incubation might favor protein diffusion into the spheroid/organoid center as previously reported for full-length immunoglobulins that per se are comparable in size to the herein developed biosensors (Beltrán Hernández et al., 2019). Finally, the herein presented experiments were carried out by using widefield or confocal microcopy to determine analyte changes in classical 2D cell culture. However, more complex imaging techniques will be required for advanced applications, such as 2-photon microscopy that allows determination of FRET changes in the center of, for instance, large spheroids or organoids.

## STAR★Methods

### Key resources table

**Table:** 

REAGENT or RESOURCE	SOURCE	IDENTIFIER
**Antibodies**		

Cy™5 AffiniPure Goat Anti-Alpaca IgG, VHH domain	Jackson ImmunoResearch	128-175-232

**Bacterial and virus strains**		

*E.coli* Arctic Express (DE3)	Agilent	#230192
*E.coli* BL21 (DE3)	Thermo Fisher Scientific	EC0114
NEB 5-alpha competent *E.coli*	New England BioLabs	C2987H

**Biological samples**		

Primary hippocampal mouse neurons	This paper	N/A

**Chemicals, peptides, and recombinant proteins**		

3-(4,5-Dimethylthiazol-2-yl)-2,5-Diphenyltetrazolium Bromide (MTT)	Thermo Fisher Scientific	M6494
Agar-Agar Kobe I	Carl Roth	
Agarose	VWR International	Cat#732-2789
Ammoniumpersulfat	Thermo Fisher Scientific	17,874
Basal Medium Eagle	Thermo Fisher Scientific	Cat#21010046
Bovine Serum Albumin (BSA)	Carl Roth	A2153
CaCl2 2H2O	Carl Roth	Cat#5239
InstantBlue Coomassie Protein Stain	Abcam	Ab119211
D-Glucose H2O	Carl Roth	Cat#6887
Digitonin	Sigma Aldrich	Cat#D141-100MG
Dimethylsulfoxid (DMSO)	Carl Roth	A994.1
DMEM+Glutamax	Thermo Fisher Scientific	11594446
DNAse I	AppliChem	A3778
Fetal bovine serum	Thermo Fisher Scientific	Cat#10270106
G418	Sigma Aldrich	Cat#A1720
Glycerin	Carl Roth	Carl Roth
HEPES	Carl Roth	Cat#9105
HER2-Nb	Thermo Genesynthesis based on Vaneycken et al., 2011	N/A
HER2-Nb-GEPII 1.0	This paper	N/A
HER2-Nb-pH-Lemon	This paper	N/A
Hoechst 33342	Thermo Fisher Scientific	Cat#62249
Imidazole	AppliChem	A3635,0100
IPTG	Carl Roth	CN08.3
Kanamycin	Carl Roth	T832.2
KCl	Carl Roth	
KCl	Carl Roth	Cat#6781
Lysozym	AppliChem	A4972
MgCl2 6H2O	Carl Roth	Cat#A537
Monarch Plasmid Miniprep Kit	New England Biolabs	Cat#T1010S
NaCl	Carl Roth	Cat#9265
Neurbasal medium	Thermo Fisher Scientific	Cat#21103049
N-Methyl-D-Glucamine (NMDG)	Sigma Aldrich	Cat#M2004
OPTI-Mem	Thermo Fisher Scientific	11524456
PBS	Thermo Fisher Scientific	11503387
PCR Mycoplasma Test Kit I/C	PromoCell	PK-CA91-1048
Penicillin/Streptomycin	Thermo Fisher Scientific	11548876
PMSF BioChemica	AppliChem	A0999
Poly-L-Lysine	Sigma Aldrich	Cat#9155
Protease-Inhibitor Mix B	Serva	39105.03
PageRuler Plus Prestained Protein Ladder	Thermo Fisher Scientific	15543197
Q5 High-Fidelity DNA Polymerase	New England Biolabs	Cat#M0491S
QIAGEN Plasmid Midi Kit	QIAGEN	Cat#12143
ROTIPHORESE Gel 30	Carl Roth	3029.1
Sodium pyruvate	Thermo Fisher Scientific	Cat#11360070
Sodiumdodecylsulfate	Carl Roth	0183.3
SPOT-Nb-ATTO488	Chromotek	N/A
SPOT-Nb-FLII	This paper	N/A
SPOT-Nb-GEPII 1.0	This paper	N/A
SPOT-Nb-pH-Lemon	This paper	N/A
SPOT-tag	Chromotek	N/A
TEMED	Carl Roth	8142.1
Terrififc-Broth-Medium	Carl Roth	X972.1
Thermo Fisher Scientific	Thermo Fisher Scientific	12566014
Tris Base	Carl Roth	4855.1
TRIS-HCl	Carl Roth	9090.3
Trypsin-EDTA (0.5%)	Thermo Fisher Scientific	Cat#15400054
TWEEN	Carl Roth	9127.1

**Experimental models: Cell lines**		

HEK293	ATCC	Cat#CRL-1573
MCF7	ATCC	Cat#HTB-22
Primary hippocampal mouse neurons	This paper	
SkBr3	ATCC	Cat#HTB-30

**Recombinant DNA**		

SPOT-Nb-ATTO488	Chromotek	
SPOT-Nb-pH-Lemon	This paper	N/A
SPOT-Nb-FLII	This paper	N/A
HER2-Nb-pH-Lemon	This paper	N/A
GPI-SPOT	This paper	N/A
HER2 WT	Addgene	#16257
HER2-Nb	Thermo Genesynthesis based on Vaneycken et al., 2011	N/A
SPOT-Nb-GEPII 1.0	This paper	N/A
HER2-Nb-GEPII 1.0	This paper	N/A

**Software and algorithms**		

BioRender	BioRender	https://biorender.com/
Corel Draw	Corel Draw Graphics Suite	https://www.coreldraw.com/de/
ImageJ	Schneider et al., 2012	https://imagej.nih.gov/ij/
MetaXpress	Molecular Devices	https://de.moleculardevices.com/
Microsoft Excel Office 365	Microsoft	https://www.office.com/
Prism 9	GraphPad	https://www.graphpad.com/ scientific-software/prism/
Visiview	Visitron Systems	https://www.visitron.de/prod ucts/visiviewr-software.html

### Resource availability

#### Lead contact

Further information and requests for resources and reagents should be directed to and will be fulfilled by the lead contact, Ulrich Rothbauer (ulrich.rothbauer@uni-tuebingen.de).

#### Materials availability

Materials reported within this study are available from the lead contact on request.

### Experimental model and subject details

#### Standard cell lines

HEK293, MCF7 and SkBr3 cells were purchased from ATCC (Virginia, US). All cell lines were cultivated at 37°C and 5% CO_2_ in a humidified incubator using DMEM (Thermo Fisher Scientific) + 10% FBS. Cells were kept in maintenance on a T75 flask by passaging at a confluency of 70–80% using trypsin-EDTA. The cell lines were tested for mycoplasma using the PCR Mycoplasma kit I/C (PromoCell). Since this study does not include cell line-specific analysis, cell lines were used without additional authentication.

#### Primary hippocampal mouse neurons

Hippocampal neurons were obtained from male and female C57BL/6N pubs at day of birth (P0). Cells were isolated according to a protocol authorized by the local Ethics Committee for Animal Research (Regierungspräsidium Tübingen, No. PZ 01/21 M) and experiments were performed in accordance with the German Animal Welfare Act and the ARRIVE guidelines for reporting animal research. Animals for breeding were maintained on a 12/12 h light/dark cycle with access to food and water *ad libitum*. Following preparation in dissection medium (HBSS with 1% sodium pyruvate, 1% HEPES (1 M) and 0.5% glucose (1 M)) hippocampal tissue was digested for 20 min in 2.5% trypsin at 37°C. After repeated washing in dissection medium remaining tissue pieces were thoroughly triturated. Dissociated neurons were seeded at a density of 110,000 cells in ibidi 12 mm Culture-Inserts (ibidi GmbH) on poly-L-Lysine coated 30 mm circular glass slides in 6-well plates. After cell attachment in plating medium (BME with 1% sodium pyruvate, 1% Glutamax, 10% FBS, 1% penicillin/streptomycin (P/S)) hippocampal neurons were cultured in maintenance medium (Neurobasal (NB) medium with 2% B27, 1% sodium pyruvate, 1% P/S) for 8 days. At DIV 5 20% (v/v) of the medium was exchanged with fresh NB medium.

#### Bacterial expression systems

*E. coli* NEB 5-alpha competent *E. coli* cells (New England Biolabs, Ipswich, MA, USA) were used for cloning procedures. For protein expression, *E. coli* Arctic Express (DE3) (Agilent Technologies, Waldbronn, Germany) and *E. coli* BL21 (DE3) (New England Biolabs, Ipswich, MA, USA) were used.

### Method details

#### Construct design and cloning

For the construction of the Nb-fused biosensors, the cDNA of SPOT-Nb (kindly provided by ChromoTek, Martinsried, Germany) and HER2-Nb 2Rs15d (Vaneycken et al., 2011) obtained as a gene synthesis product from Thermo Fisher (Schwerte, Germany) were used. The biosensor moieties used were the GEPII 1.0 potassium sensor (Bischof et al., 2017), the pH reporter pH-Lemon (Burgstaller et al., 2019), and the glucose sensor FLII12Pglu700μd6 (further referred to as FLII) (Takanaga et al., 2008). GEPII 1.0 consists of mseCFP, the potassium-binding domain (Kbp), and cpV, reporting K^+^ alterations by a conformational change of the Kbp (Bischof et al., 2017). Within pH-Lemon, the pH stable mTurquoise2 is directly connected to the pH-sensitive EYFP via a flexible linker and responds to pH changes because of the differential pH sensitivities of the mTurquoise2 (pKa 3.1) and EYFP (pKa 6.9) (Burgstaller et al., 2019; Goedhart et al., 2012; Ormö et al., 1996). Within FLII, the FRET donor ECFP is flanked by parts of the glucose-binding domain MglB on each end, whereas the FRET acceptor is located on the C-terminal end (Deuschle et al., 2005; Takanaga et al., 2008).

All expression constructs of Nb-fused biosensors were designed with the Nb at the N- and the sensor moiety at the C-terminus. Therefore, the cDNA of the Nbs were genetically fused to the sensor thereby including a flexible (Gly_4_Ser)_4_ linker. Subsequently, SPOT-Nb- and HER2-Nb- containing constructs were cloned into a pET28a(+) vector (Merck-Millipore, Darmstadt, Germany), thereby adding a C-terminal hexahistidine tag (His_6_-tag) using conventional, PCR and restriction enzyme-based cloning. Finale expression constructs were subcloned in NEB 5-alpha competent *E. coli* cells (New England Biolabs, Ipswich, MA, USA) followed by Sanger sequencing (Microsynth, Göttingen, Germany).

#### Protein expression using bacterial expression systems

To achieve a high fraction of soluble biosensors SPOT-Nb-GEPII 1.0, SPOT-Nb-pH-Lemon, SPOT-Nb-FLII, HER2-Nb-GEPII 1.0, and HER2-Nb-pH-Lemon were transformed into chemically competent *E. coli* Arctic Express (DE3) (Agilent Technologies, Waldbronn, Germany) following the manufacturer's guidelines. Briefly, a 50 mL over-night culture containing kanamycin 25 μg/mL (Carl Roth GmbH, Karlsruhe, Germany) and G418 (Sigma Aldrich, Schnelldorf, Germany) was inoculated with one single colony. The overnight culture was transferred to 0,2 L main culture resulting in a starting OD_600_ of 0.2. Protein expression was induced at an OD_600_ of 0.8 using 0.5 mM IPTG (Carl Roth GmbH) followed by incubation for 24 h at 12°C. Subsequently, the cells were harvested (6000 x g, 10 min, 4°C), resuspended in IMAC binding buffer (15 mM imidazole, 500 mM NaCl, pH 7.6 in PBS) containing 2 mM phenylmethylsulfonyl fluoride (PMSF, AppliChem GmbH, Darmstadt, Germany), flash-frozen in liquid nitrogen and stored at −80°C. For the production of HER2-Nb, the expression construct was transformed into *E. coli* BL21 (DE3) following the manufacturer’s instructions. Briefly, one single colony was used to inoculate a 50 mL overnight culture containing 100 μg/mL ampicillin. The overnight culture was transferred to the 1 L main culture resulting in a starting OD_600_ of 0.2. Protein expression was induced at an OD_600_ of 0.8 using 1 mM IPTG followed by incubation for 20 h at RT. Cells were resuspended in IMAC binding buffer (15 mM imidazole, 500 mM NaCl, pH 7.6 in PBS) after harvesting (6000 x g, 10 min, 4°C) containing 2 mM PMSF, flash-frozen in liquid nitrogen and stored at −80°C.

#### Protein purification

For protein purification, the bacterial cells were thawed and 0.16 mg/mL lysozyme (AppliChem GmbH, Darmstadt, Germany), 0.16 mg/mL DNAseI (Thermo Fisher Scientific, Waltham, MA, USA) and protease inhibitor mix B (SERVA Electrophoresis GmbH, Heidelberg, Germany) were added, followed by 2 rounds (15 min each) of sonication, interrupted by 60 min incubation in a rotary shaker at 4°C. After harvesting (45 min, 18000 x g, 4°C), the supernatant of Nb-biosensors was collected, and the pellet was dissolved in 2 mL of 2 M imidazole to extract residual proteins. After sonication (4 × 30 s) and harvesting (45 min, 18000 x g, 4°C), both supernatants were combined, filtered (0.45 μm) and diluted with 500 mM NaCl in PBS to a final imidazole concentration of 15 mM and a pH of 7.6. The solution was loaded onto a 5 mL HisTrap column (Cytiva, MA, USA), washed with 20 column volumes of washing buffer (15 mM imidazole, 500 mM NaCl, pH 7.6 in PBS), followed by protein elution (500 mM imidazole, 500 mM NaCl, pH 7.4 in PBS) of individual fractions. Fractions showing high amounts of the target protein and low impurities (as determined using SDS-PAGE) were combined and concentrated using an Amicon concentrator tube (30 kDa MWCO for the Nb-fused biosensors, 3 kDa MWCO for HER2-Nb) (Merck-Millipore). A final volume <5 mL was loaded onto a SEC column (HiLoad 200pg, 16&600 for Nb-fused biosensors; HiLoad 75pg, 26&600 for HER2-Nb) (Cytiva) and eluted with HEPES buffer (10 mM HEPES, 150 mM NaCl, pH 7.4 in ddH_2_0). SEC fractions containing full length proteins were concentrated to a concentration of ∼1 mg/mL as determined using NanoDrop (ThermoFisher). After the addition of glycerol to a final concentration of 2% (v/v), proteins were aliquoted, flash frozen and stored at −80°C until usage.

For quality control, all purified proteins were analyzed via SDS-PAGE according to standard procedures. Therefore, protein samples were denaturized (5 min, 95°C) in 2x SDS-sample buffer containing 60 mM Tris/HCl, pH 6.8; 2% (w/v) SDS; 5% (v/v) 2-mercaptoethanol, 10% (v/v) glycerol, 0.02% bromphenole blue. All proteins were visualized by InstantBlue Coomassie (Expedeon) staining. For immunoblotting, proteins were transferred to nitrocellulose membrane (GE Healthcare, Chicago, IL, USA) and detection was performed using Cy-5 labelled anti-VHH antibody (Cy™5 AffiniPure Goat Anti-Alpaca IgG, VHH domain, Jackson ImmunoResearch, UK) and a Typhoon Trio scanner (GE-Healthcare, excitation 633 nm, emission filter settings 670 nm BP 30). An estimate of the proportion of full-length protein was performed by densitometric analysis of Coomassie-stained SDS-PAGE.

#### BioLayer interferometry

To determine the binding affinity of purified SPOT-fused biosensors, biolayer interferometry (BLI) was performed on an Octet device (Sartorius GmbH, Germany) according to the manufacturer’s guidelines. Briefly, biotinylated SPOT peptide (Intavis, Tuebingen, Germany) loaded onto Streptavidin-coated sensor tips. With different concentrations of SPOT-Nb-GEPII 1.0 and SPOT-Nb-pH-Lemon (6.25, 12.5, 25 and 50 nM) and SPOT-Nb-FLII (12.5, 25, 50 and 100 nM) diluted in Octet buffer (0.1% BSA (v/v), 0.02% Tween 20 (v/v) in PBS) four association/dissociation runs were performed to determine the association and dissociation rate constant.

#### Cells seeding and preparation for fluorescence microscopy

For fluorescence widefield microcopy HEK293 cells were cultivated on 30 mm circular glass slides, while cells were seeded into 96-well imaging plates (Greiner, Kremsmünster, Austria) for confocal microscopy experiments. For immobilization of SPOT-Nb-sensors on HEK293 cells, the cells were transfected with a plasmid encoding a GPI-anchored SPOTtag (GPI-SPOT) using Lipofectamine 2000 reagent (Thermo Fisher) according to the manufacturer's instructions 2 days before experiments, followed by the removal of the transfection reagent after 6 h. For transient HER2 overexpression in HEK293 cells, the cells were transfected with a plasmid encoding human HER2 wildtype (Li et al., 2004)(Addgene plasmid #16257) the day before measurement with removal of the transfection reagent after 6 hours. Neuronal cultures were transfected with GPI-SPOT using lipofectamine 0.32 μL and DNA 1.6 μg diluted in 160 μL OPTI-MEM (2x80 μL). 160μL culture medium (from 0.5 mL total volume) was removed and 160 μL of the transfection mix was added. On two consecutive days (24 and 48 h after the start of transfection), 3/4 of the medium was replaced to dilute the transection mix. Cells were analyzed 5 days after transfection.

#### Immobilization of Nb-biosensors

For immobilization of SPOT-Nb-GEPII 1.0, SPOT-Nb-pH-Lemon, SPOT-Nb-FLII, HER2-Nb-GEPII 1.0 and HER2-Nb-pH-Lemon, the proteins were diluted to a final concentration of 2 μM in a physiological buffer (138 mM NaCl, 5 mM KCl, 2 mM CaCl_2_, 1 mM MgCl_2_, 10 mM D-glucose, 10 mM HEPES, pH 7.4 in ddH_2_0) to a final volume of 1 mL (for 6–well format) or 0.2 mL (for 96-well format). Cells were washed once with PBS and incubated with the Nb-based biosensor solution for 20–30 min at RT in the dark. After immobilization, the cells were washed three times with physiological buffer and immediately used for measurements.

#### Fluorescence microscopy

For high-resolution fluorescence microscopy an ImageXpress MicroConfocal Microscope (Molecular Devices, California, US) equipped with a 40X water immersion objective was used. Cells were imaged in a physiological buffer, acquiring DAPI, mseCFP/mTurquoise2 and cpV/EYFP images. For FRET-based live-cell widefield imaging experiments, a Zeiss Axio Observer Z1 equipped with a 40x oil immersion objective (EC “Plan-Neofluar” 40x/1,30 Oil M27) (Zeiss, Oberkochen, Germany) and connected to an OptoSplit II emission image splitter (Cairn Research, Faversham, UK) equipped with a T505lpxr (AHF Analysentechnik, Tübingen, Germany) was used. For illumination, an LedHUB LED light-engine equipped with a 455 nm LED and a 505–600 nm LED (Omicron, Dudenhofen, Germany) with 427/10 nm and 510/10 nm excitation filters (AHF Analysentechnik) was employed. Dichroic and emission filter in the microscope were as follows: 459/526/596 and 475/543/702 (AHF Analysentechnik). Image acquisition and microscope control were performed using VisiView software (Visitron Systems GmbH, Puchheim, Germany). For buffer exchange, a gravity-based perfusion system (NGFI GmbH, Graz, Austria) was connected to a PC30 perfusion chamber (NGFI GmbH).

#### Functional characterization of Nb-fused biosensors

For the titration of different K^+^, pH and glucose concentrations, the physiological buffer was modified accordingly. Briefly, buffers for K^+^ titrations consisted of 2 mM CaCl_2_, 1 mM MgCl_2_, 10 mM HEPES, 10 mM glucose, pH 7.4 with different concentration of KCl to obtain 0, 0.3, 1, 3, 10, 30, and 100 mM K^+^. To maintain buffer osmolarity, the concentration of NaCl was adjusted. Buffers for pH titrations consisted of physiological buffers pH values were adjusted using N-Methyl-D-glucamin (NMDG) or HCl to obtain pH 4, 5, 6, 7, 7.5, 8 and 9. Buffers for glucose titrations consisted of physiological buffer. Different glucose concentrations were used to obtain 0, 0.1, 0.3, 1, 3, 10, 30 and 100 mM glucose. Delta ratio values were obtained by subtracting the FRET ratio values at 0 mM K^+^, 0 mM glucose or pH 5.0 (= minimal FRET ratio) from all individual FRET ratio values obtained in response to distinct K^+^, glucose or pH alterations (R-R_min_), thereby setting the minimal FRET values to 0.

#### Visualization of neuronal K^+^ release

For visualization of K^+^ release, hippocampal neurons growing on 30 mm circular glass slides were inserted into the PC30 perfusion chamber and connected to the perfusion system (NGFI GmbH). Experiments were performed using the Zeiss Axio Observer Z1 microscope. 2 μM SPOT-Nb-GEPII 1.0 was immobilized on the cell surface for 30 min in physiological buffer. To remove unbound sensor protein after immobilization, the cells were shortly perfused with K^+^-free buffer. Subsequently, the perfusion was stopped, and 50 μL of the pure buffer without glutamate was added by pipetting, followed by a glutamate bolus (50 μL, 10 μM final concentration). Following the K^+^ increase upon glutamate addition, the perfusion was started to remove extracellular K^+^ using K^+^-free physiological buffer, followed by perfusion with a 100 mM K^+^ buffer to determine the maximal sensor response.

#### Determination of cell viability

Cell viability over time of control cells (no incubation with Nb) and after immobilization of HER2-Nb and HER2-Nb-GEPII 1.0 was assessed using 3-(4,5-Dimethylthiazol-2-yl)-2,5-Diphenyltetrazolium Bromide (MTT) reagent (Thermo Fisher). After immobilization of HER2-Nb and HER2-Nb-GEPII 1.0 on the cell surface, cells were cultivated for either 3, 6, 24 or 48 h in DMEM +10% FBS before the assay. MTT assay was performed according to manufacturer’s instructions. Absorbance at 540 nm was recorded using a Tecan Infinite 200 PRO plate reader. For all time-points, cells without immobilized HER2-Nb or HER2-Nb-biosensor served as a 100% viability control.

#### Visualization of K^+^ release from necrotic cells

SkBr3 cells were seeded on 30 mm circular glass coverslips and HER2-Nb-GEPII 1.0 was added for immobilization on endogenous HER2. After immobilization and washing, the coverslip was inserted in the PC30 chamber and 0.95 mL of a 5 mM K^+^ buffer was added. While recording the FRET ratio by acquiring the FRET and CFP fluorescence emissions, 50 μL of a 200 μM digitonin stock solution was added by pipetting to achieve a final concentration of 10 μM. Brightfield images were acquired from SkBr3 cells in response to digitonin to monitor morphological changes.

### Quantification and statistical analysis

Data analysis was performed using Excel (Microsoft, NM, USA), MetaXpress (Molecular Devices, San Jose, CA, USA) and ImageJ (Schneider et al., 2012). Statistical analysis was performed using GraphPad Prism 9 software (GraphPad Software, San Diego, CA, USA). Data were analyzed for normal distribution using D’Agostino–Pearson omnibus normality test. p values <0.05 were considered significant. Statistical tests used for the analysis of respective panels are indicated in the figure legend. Illustrations (Figures 3A and 5A) were created with BioRender.com.

## Data Availability

• All data reported in this paper will be shared by the lead contact on request. • This paper does not report original code. • Any additional information required to reanalyze the data reported in this paper is available from the lead contact on request.
