# Supplementary material for: Monitoring extracellular ion and metabolite dynamics with recombinant nanobody-fused biosensors
Source: iScience. 2022 Aug 10;25(9):104907. doi: 10.1016/j.isci.2022.104907 (PMC9421384; doi:10.1016/j.isci.2022.104907)
Supplement: Document S1. Figures S1–S7 and Tables S1 [file mmc1.pdf]

iScience, Volume *25*

Supplemental information

Monitoring extracellular ion

and metabolite dynamics

with recombinant nanobody-fused biosensors

Sandra Burgstaller, Teresa R. Wagner, Helmut Bischof, Sarah Bueckle, Aman Padamsey, Desiree Frecot, Philipp D. Kaiser, David Skrabak, Roland Malli, Robert Lukowski, and Ulrich Rothbauer

Supplementary Figures:

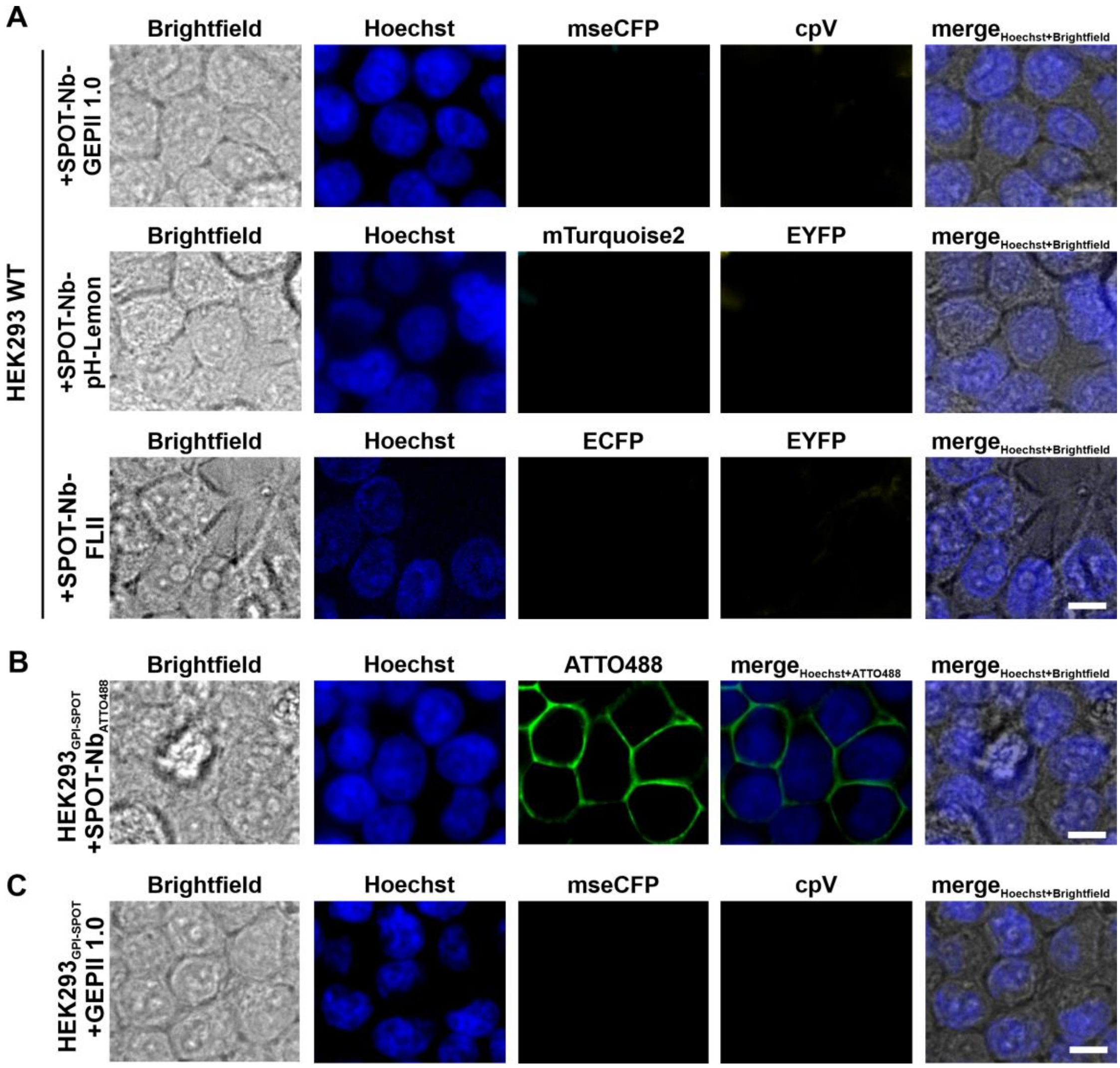

Figure S1 (related to Figure 1). SPOT-Nb-biosensors do not bind unspecifically to the plasma membrane. Representative confocal images of (**A**) live HEK293 wildtype (WT) cells following incubation with SPOT-Nb-GEPII 1.0 (upper panel), SPOT-Nb-pH Lemon (middle panel) and SPOT-Nb-FLII (lower panel. (**B**) Live imaging of HEK293 cells expressing GPI-anchored SPOT-tag (GPI-SPOT) on the plasma membrane after staining with the fluorescently labeled SPOT-Nb (SPOT-Nb$_{ATTO488}$) and (**C**) of live HEK293 GPI-SPOT cells upon incubation with GEPII 1.0 biosensor lacking the SPOT-Nb. (**A – C**) Scale bar 10 µm, n= 4 experiments for (**A**, upper & middle row) and (**B**); n= 2 experiments for (**A**, lower row) and (**C**).

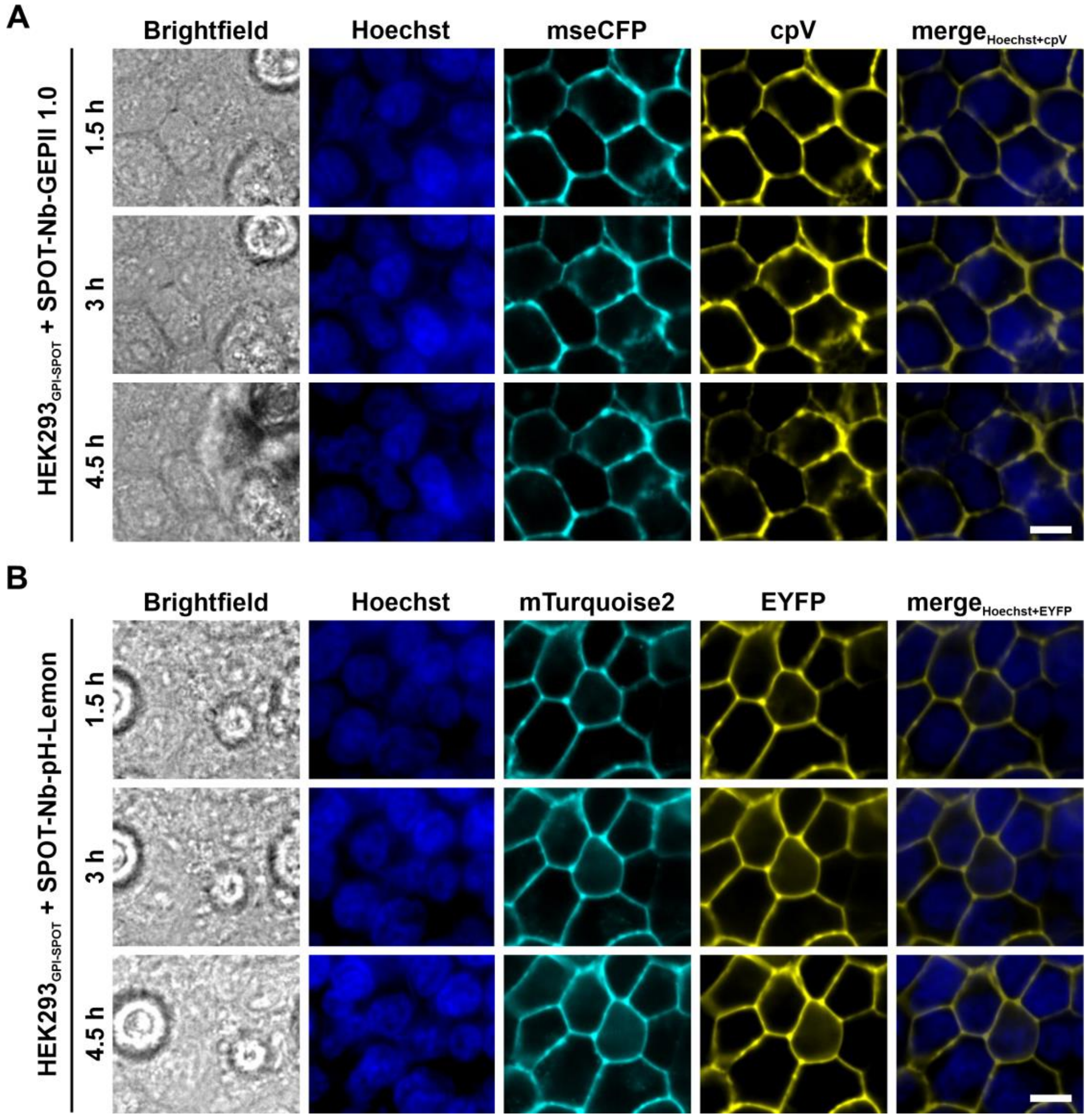

Figure S2 (related to Figure 1). Long term binding of SPOT-Nb-biosensors to the plasma membrane of living cells. Time-lapse confocal microscopy of HEK293 GPI-SPOT expressing cells following incubation with SPOT-Nb-GEPII 1.0 (**A**) or SPOT-Nb-pH-Lemon (**B**). Representative images of n=4 experiments at indicated time points are shown. Scale bar 10 µm.

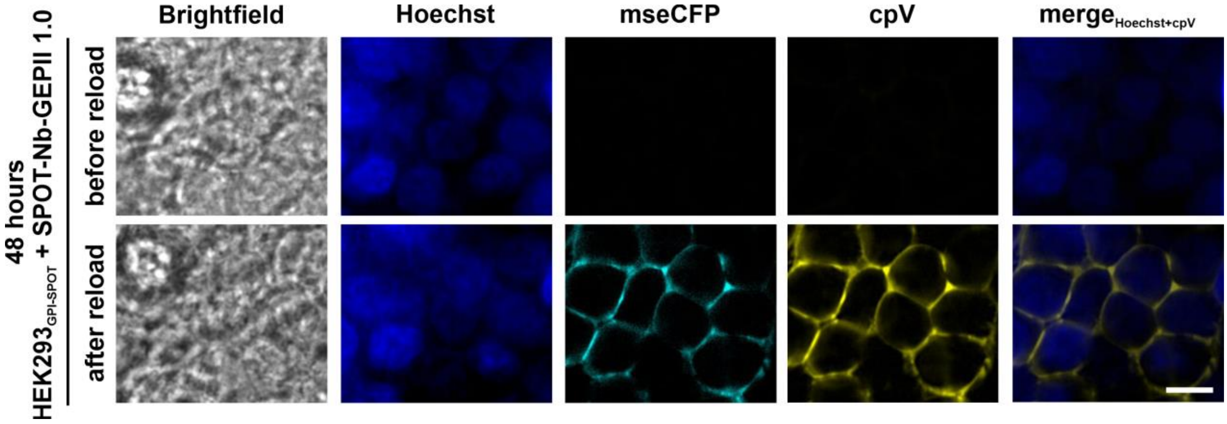

Figure S3 (related to Figure 1). Re-loading of the GPI-SPOT anchor with SPOT-Nb-GEPII 1.0 at the plasma membrane. Representative confocal images of HEK293 GPI-SPOT cells 48 h after incubation with SPOT-Nb-GEPII 1.0 (upper row) or upon re-loading of SPOT-Nb-GEPII 1.0 (lower row). Scale bar 10 µm.

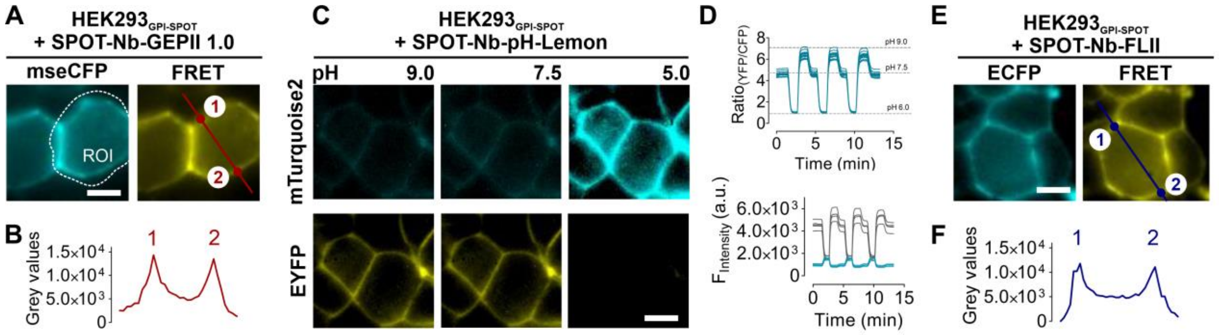

Figure S4 (related to Figure 2). Widefield FRET-imaging of SPOT-Nb-biosensors. (**A**) Representative widefield images of HEK293 GPI-SPOT cells displaying the mseCFP (left panel) and the FRET signal (right panel) derived from SPOT-Nb-GEPII 1.0 binding. An exemplary region (ROI, white dotted line) used for measuring the FRET response is indicated. (**B**) Intensity profile of a line scan (red) crossing the plasma membrane. Peaks (1 and 2) in (**B**) correspond with the respective labelling of FRET image in (**A**). (**C**) Representative widefield images of HEK293 GPI-SPOT cells displaying either mTurquoise2 (upper row) or EYFP signal (lower row) derived from bound SPOT-Nb-pH-Lemon in response to alkaline pH (9.0), neutral pH (7.5) and acidic pH (5.0). (**D**) Ratio signals of individual cells in response to repetitive pH alterations (switching between pH 7.5, 6.0, and 9.0, upper panel). Respective single wavelength traces (EYFP, grey and mTurquoise2, blue) upon pH alterations (lower panel). (**E**) Representative widefield images of HEK293 GPI-SPOT cells displaying ECFP (left panel) or FRET signal (right panel) derived from bound SPOT-Nb-FLII. (**F**) Intensity profile of a line scan (blue) crossing the plasma membrane. Peaks (1 and 2) in (**F**) correspond with the respective labelling of FRET image in (**E**). (**A,C,E**) Scale bar 10 µM, (**A,C,D,E**) n= 3 experiments from biological replicates.

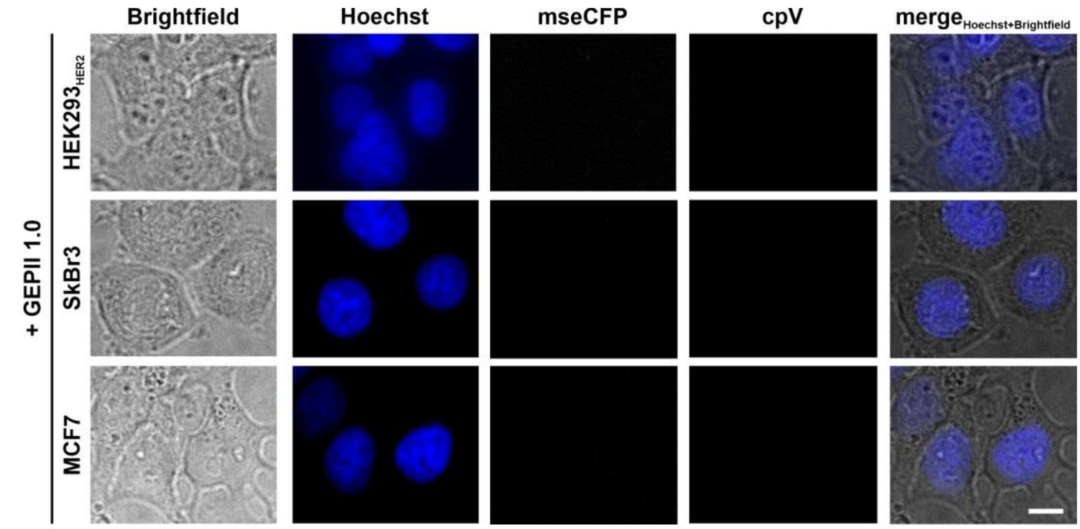

Figure S5 (related to Figure 4 and Figure 5). Purified GEPII 1.0 lacking the HER2-Nb does not bind to the cell surface of cancer cells. Representative confocal live imaging of HEK293 cells transiently overexpressing HER2 ($HEK293_{HER2}$, upper row), SkBr3 cells (middle row) and MCF7 cells (lower row) after addition of purified GEPII 1.0 lacking the HER2-Nb are shown. Scale bar 10 µm, n= 2 experiments.

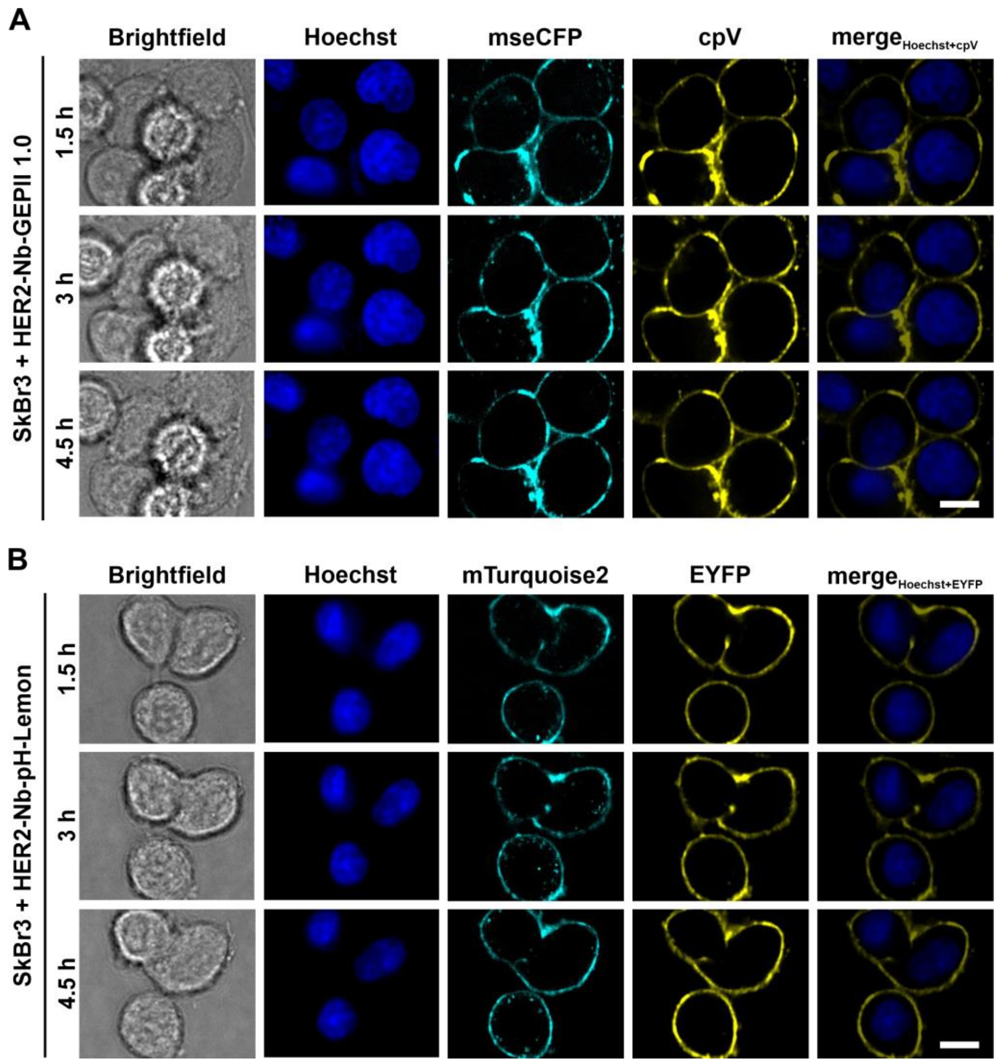

Figure S6 (related to Figure 5). Long term binding of HER2-Nb-biosensors to the plasma membrane of living cancer cells. Time-lapse confocal microscopy of SkBr3 cells following incubation with HER2-Nb-GEPII 1.0 (**A**) or HER2-Nb-pH-Lemon (**B**). Representative images at indicated time points are shown. Scale bar 10 µm, n= 2 experiments.

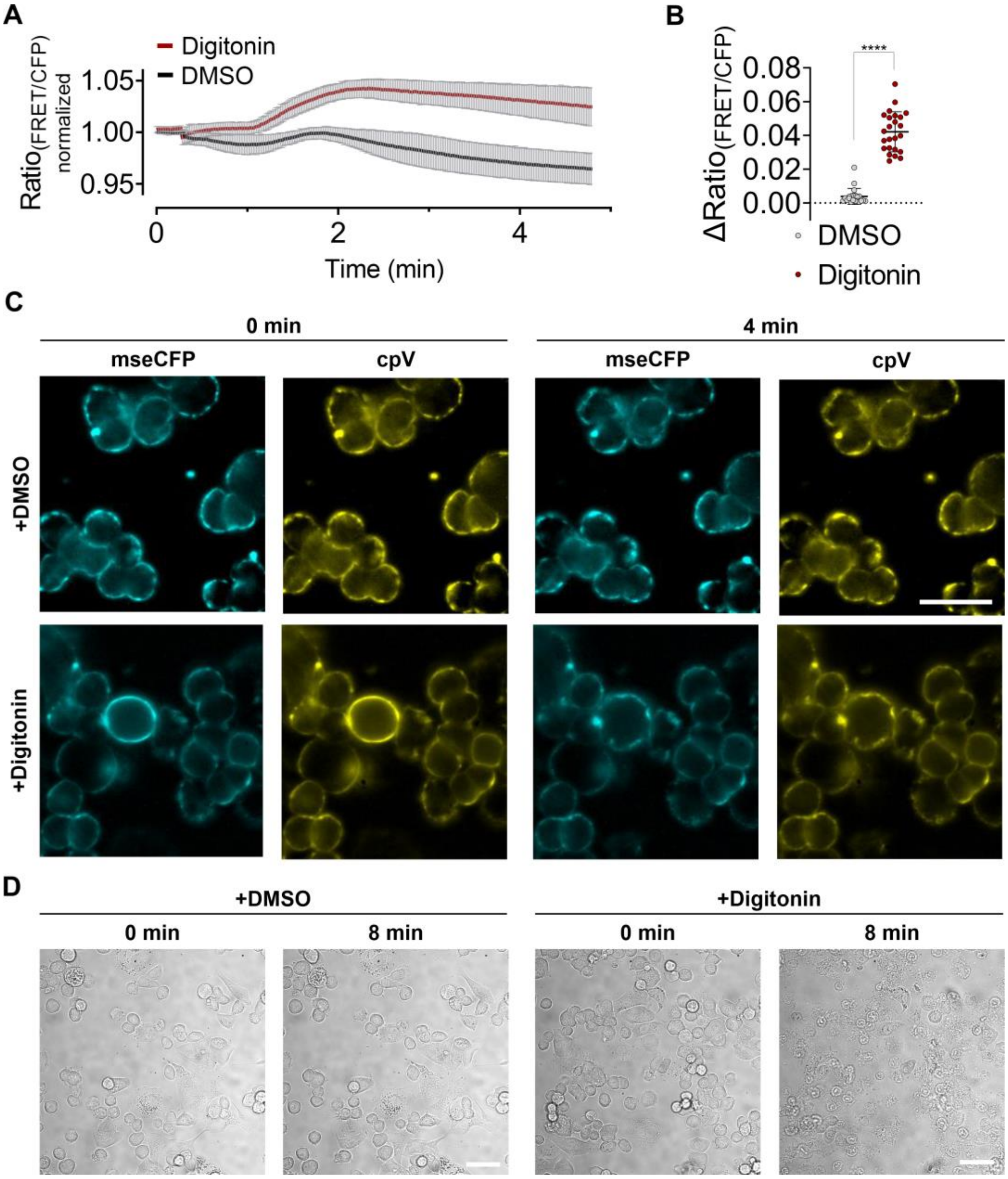

Figure S7 (related to Figure 5). Dynamic measurements of digitonin-induced K^+-release. (**A**) FRET ratio traces of HER2-Nb-GEPII 1.0 (mean ± SD) immobilized on SkBr3 cells in response to DMSO (black, n=2, N= 21) and 10 µM digitonin (red, n=2, N= 24). (**B**) Maximal FRET ratio changes displayed as ΔRatio ($R-R_{min}$) of SkBr3 cells in response to DMSO or digitonin. $p < 0.0001$, Mann Whitney nonparametric t-test. (**C**) Fluorescence widefield images showing mseCFP and FRET of immobilized HER2-Nb-GEPII 1.0 at starting conditions (0 min) and after 4 minutes (4 min) of either DMSO (upper row) or digitonin (lower row) treated cells. (**D**) Widefield images displaying cell death of SkBr3 cells before (0 min) and after (8 min) DMSO (left panels) and digitonin (right panels) treatment (n= 3). Scale bars represent 50 µm.

Table S1 (related to Figure 1): Binding affinities of SPOT-Nb-biosensors determined using biolayer interferometry.

**Nbs**	**K_D [nM]**	**k_{on} [10^5 M^{-1} s^{-1}]**	**k_{off} [10^3 s^{-1}]**
SPOT-Nb-GEPII 1.0	1.04 ± 0.01	0.43 ± 0.002	0.04 ± 0.0003
SPOT-Nb-pH-Lemon	4.74 ± 0.02	0.65 ± 0.002	0.31 ± 0.0003
SPOT-Nb-FLII	14.9 ± 0.21	0.18 ± 0.002	0.26 ± 0.0003
